# Modeling cortical synaptic effects of anesthesia and their cholinergic reversal

**DOI:** 10.1371/journal.pcbi.1009743

**Published:** 2022-06-23

**Authors:** Bolaji P. Eniwaye, Victoria Booth, Anthony G. Hudetz, Michal Zochowski

**Affiliations:** 1 Department of Applied Physics, University of Michigan, Ann Arbor, Michigan, United States of America; 2 Department of Mathematics and Department of Anesthesiology, University of Michigan, Ann Arbor, Michigan, United States of America; 3 Center for Consciousness Science, Department of Anesthesiology, University of Michigan, Ann Arbor, Michigan, United States of America; 4 Department of Physics and Biophysics Program, University of Michigan, Ann Arbor, Michigan, United States of America; National Research Council, ITALY

## Abstract

General anesthetics work through a variety of molecular mechanisms while resulting in the common end point of sedation and loss of consciousness. Generally, the administration of common anesthetics induces reduction in synaptic excitation while promoting synaptic inhibition. Exogenous modulation of the anesthetics’ synaptic effects can help determine the neuronal pathways involved in anesthesia. For example, both animal and human studies have shown that exogenously induced increases in acetylcholine in the brain can elicit wakeful-like behavior despite the continued presence of the anesthetic. However, the underlying mechanisms of anesthesia reversal at the cellular level have not been investigated. Here we apply a computational model of a network of excitatory and inhibitory neurons to simulate the network-wide effects of anesthesia, due to changes in synaptic inhibition and excitation, and their reversal by cholinergic activation through muscarinic receptors. We use a differential evolution algorithm to fit model parameters to match measures of spiking activity, neuronal connectivity, and network dynamics recorded in the visual cortex of rodents during anesthesia with desflurane *in vivo*. We find that facilitating muscarinic receptor effects of acetylcholine on top of anesthetic-induced synaptic changes predicts the reversal of anesthetic suppression of neurons’ spiking activity, functional connectivity, as well as pairwise and population interactions. Thus, our model predicts a specific neuronal mechanism for the cholinergic reversal of anesthesia consistent with experimental behavioral observations.

## Author summary

Here, we apply a computational model of a network of excitatory and inhibitory neurons to simulate the effects of changes in synaptic inhibition and excitation due to anesthesia and to investigate the possibility of their reversal by muscarinic receptor activation. Specifically, we use a differential evolution algorithm to fit model parameters to match dynamics recorded in the visual cortex of rodents during anesthesia with desflurane *in vivo*. We find that changes of the fitted synaptic parameters in response to increasing anesthetic concentration mirrored those found in neurophysiological experiments. Further, our results demonstrate that the neuronal network effects induced by anesthesia can be mitigated by the increases in cellular excitability due to the acetylcholine mediated M-current.

## Introduction

**Fig 1:**
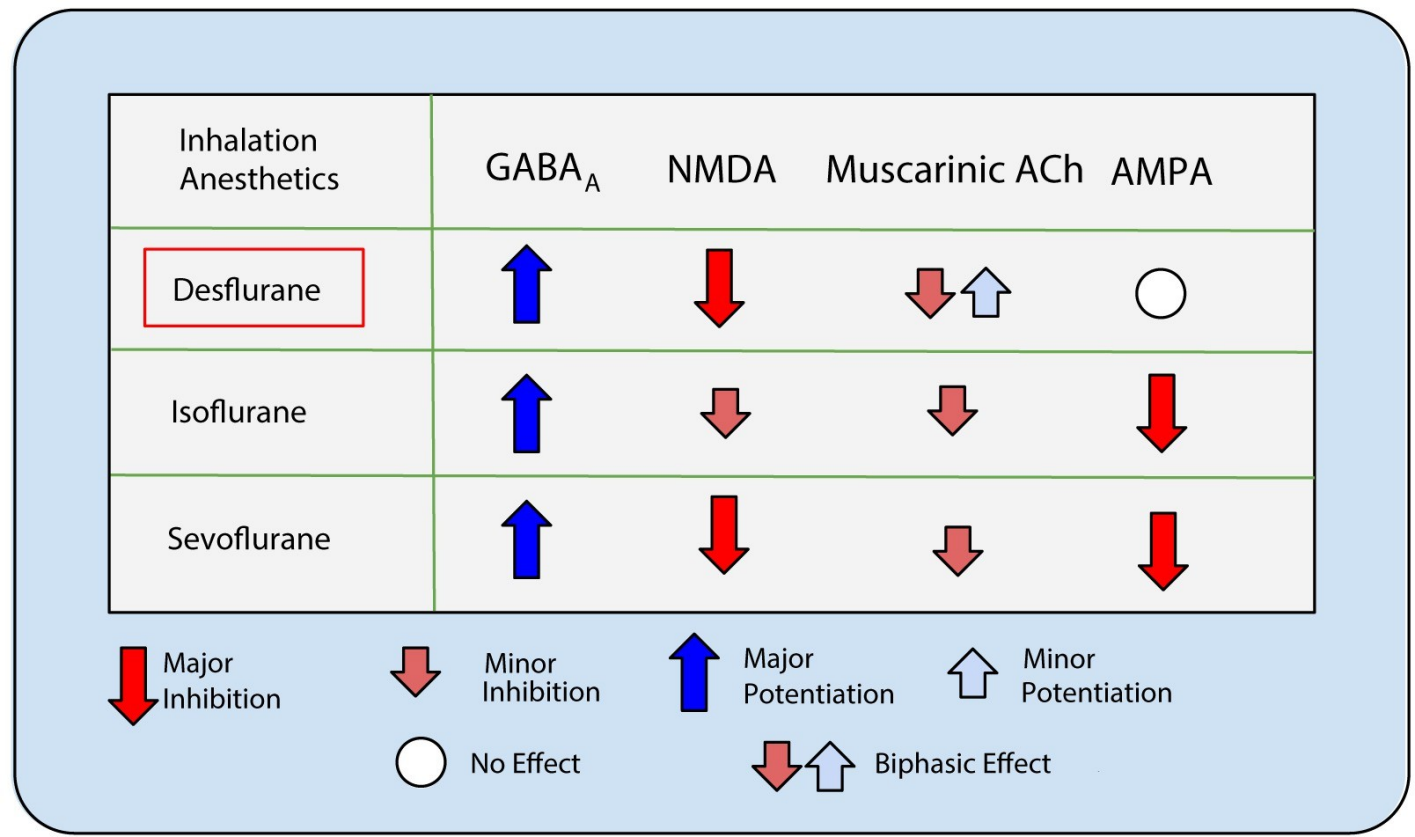
Common inhalation anesthetics have similar effects on synaptic receptors. Experimental findings show similar effects across inhalation anesthetics on synaptic receptors [29,30,38,92]. Binding to inhibitory GABA_A_ receptors is commonly potentiated while NMDA receptor activity is commonly inhibited with the magnitude of effect varying between anesthetics. Activation of muscarinic acetylcholine receptors and AMPA receptors is inhibited by isoflurane and sevoflurane while desflurane has a biphasic effect and null effect on muscarinic acetylcholine and AMPA receptors, respectively.

General anesthesia is a pharmacological procedure that is used extensively in the medical profession. The goal of anesthesia is typically to suppress the patient’s conscious awareness, stress and pain associated with surgery. Several putative mechanisms have been proposed as to how anesthetic agents induce loss of awareness or consciousness, however, the variety of effects of different anesthetic agents within the central nervous system make this an active area of study. Experimental studies implicate the brainstem, thalamus, and cortex as regions where neuronal activity is heavily modified by general anesthesia [1,2]. However, the primary target region likely depends on the type of anesthetic [3]. At the single cell level, common inhalational anesthetics facilitate inhibitory transmission and suppress excitatory synaptic transmission [4,5] while the extent of effects on specific receptors varies across different anesthetics (Fig 1). Typical inhalational anesthetics such as, isoflurane, sevoflurane and desflurane facilitate inhibitory neurotransmission at GABA_A_ receptors [6,7]. Additionally, depressive modulation of NMDA glutamate receptors has been observed for desflurane and other anesthetic agents [8,9]. The effects of most inhalational anesthetics on inhibitory and excitatory neurotransmitters have been well characterized [10,11].

Despite the differences in direct effects of different anesthetic agents, the underlying implicit hypothesis is that their final, systems-level effect leading to loss of consciousness is mediated by an anesthetic agent-invariant mechanism. Proposed neural mechanisms include modulation of neuronal excitability, increased network synchrony [12], disrupted functional connectivity and diminished information integration [13,14]. Integrated Information Theory is one of the leading theories providing an axiomatic and mathematical framework for how consciousness can be attributed to specific properties of an information processing system and how consciousness may fade when such properties are altered [15]. Indeed, experimental studies have shown that various information theoretic metrics of brain activity are reduced during anesthesia associated with suppressed behavioral signs of consciousness [16]

In order to understand the causal mechanisms of anesthetic action, additional experimental manipulations have been performed to modulate the state of consciousness. For example, pharmacological, electrical, and optogenetic stimulation of various brain regions have been performed to counter or reverse the unconscious state in humans and animals under the continued presence of anesthetic [17–20]. Many of these investigations utilized nicotinic [21] or muscarinic [22] cholinergic interventions. Recently, reverse dialysis delivery of the acetylcholine agonist carbachol was used to successfully reverse the effect of sevoflurane in rats *in vivo* [23]. Likewise, bathing cortical slices with cholinergic and noradrenergic agonists led to a reversal of stereotypical slow wave oscillations generally induced by anesthesia [24]. Nevertheless, a dissociation between behavioral and large-scale electrocortical effects of cholinergic activation in anesthetized rodents has generated some ambiguity regarding the neuronal correlates of exogenously induced anesthesia reversal [25,26]. Importantly, the question whether electrophysiological activity at cellular level in local neuronal networks is restored to pre-anesthetic levels and quality by such exogenous interventions has not been investigated. In other words, how do cortical neuronal activity patterns compare before anesthesia, during anesthesia and during the wake-like behavior elicited by exogenous stimulation while still in the presence of the anesthetic? Answering this question would not only help reveal the cellular mechanisms that may underlie the induced emergence from anesthesia but could also illuminate the synaptic pathways participating in the mechanism of anesthesia itself. In addition, the findings may have translational significance in helping to establish clinical strategies to accelerate the recovery of consciousness and cognition in surgical patients and thus ameliorate the likelihood for post-operative confusion, delirium or cognitive dysfunction including delayed emergence from anesthesia, especially in those of increased age or with prior neurological disorders [27,28]. In fact, a synaptic basis for such disorders have been proposed as many anesthetics interact through similar synaptic mechanisms [27].

In the absence of experimental measurements of single unit and cellular network activity during pharmacologically induced anesthesia reversal, computer simulation of anesthetic effects and their modulation in a neuronal network model presents a useful and promising approach. Here we embarked on such an investigation. First, we analyzed how simulated single-cell synaptic effects of the common, clinically used volatile anesthetic desflurane translate into mesoscale changes in population dynamics that have been previously recorded in the rodent visual cortex *in vivo*. In modeling desflurane we aim to study synaptic mechanisms of action common to other frequently used inhalational anesthetics including isoflurane and sevoflurane [29,30]. We then investigated how these changes may be reversed by cholinergic activation, simulated by muscarinic receptor-mediated effects on the M-current, a well-modeled K^+^ membrane current that experiences dynamical changes under cholinergic stimulation. Acetylcholine acts through multiple mechanisms and can, for example, act through the influence of nicotinic acetylcholine receptors as well as muscarinic acetylcholine receptors [31–33]. Although a focus on the muscarinic acetylcholine receptor doesn’t account for the full effects of anesthesia, previous works suggest that the muscarinic pathway has a strong identifiable role in cholinergic influence on the cortex [22]. To model this phenomenon, we simulated an excitatory-inhibitory (E-I) neuron network consisting of biophysical model neurons with glutamatergic, GABAergic and cholinergic inputs to model the effects of desflurane, a common inhalation anesthetic, by varying the effect of excitatory and inhibitory neurotransmitters in a manner consistent with experimentally observed effects of desflurane at the synaptic level. To fit the model to experimentally obtained measures of *in vivo* visual cortex network firing activity at different concentrations of desflurane, we applied a differential evolution algorithm to optimize parameters modulating the effect of neurotransmitter binding at different receptors. Specifically, we quantified the graded, concentration-dependent effect of simulated anesthetic on neuronal firing rate distributions, phase coherence, monosynaptic spike transmission, network functional connectivity, and information theoretic measures of neuronal interactions, and fit these measures to corresponding experimental data. We then used the model to simulate the presumed effect of cholinergic activation, without changing parameters for the simulated anesthetic-induced synaptic alterations, to see if these measures were reversible to near pre-anesthetic levels. Our model results provide insight into the mechanisms by which distinct neurotransmitter systems shape network behavior under the combined influence of complex pharmacological interventions that may affect the state of consciousness.

## Results

We constructed a reduced, biophysical, neuron network model to investigate how synaptic-level changes caused by the anesthetic desflurane affect network-level dynamics compared to data measured in the rodent visual cortex *in vivo*, and, separately, how muscarinic receptor-mediated changes at the cellular level may reverse these anesthetic effects. The network consisted of excitatory and inhibitory neurons interacting via synapses mediated by excitatory AMPA and NMDA receptors and inhibitory GABA_A_ receptors (see Methods section). Acetylcholine (ACh) neuromodulation of the excitability of excitatory cells was simulated as a muscarinic receptor-mediated variation in the conductance of the slow, hyperpolarizing K^+^ M-current.

We used an evolutionary algorithm (see Methods section) to identify optimal synaptic connectivity parameter sets (Tables 1 and 2) that most closely match multiple quantitative measures of network activity recorded under different desflurane concentrations. This allowed us to objectively find two sets of parameter modifications that fit model results to the experimental data. Namely, in one set of optimized parameters, we allowed the algorithm to optimize the inhibitory GABA_A_ connectivity strength and excitatory NMDA connectivity strength while keeping AMPA connectivity strength constant as simulated anesthetic concentration was increased (Tables 1 and 2, A-Series). In the second set, in addition to varying the above parameters, we allowed the M-current conductance to vary with simulated anesthetic concentration (Tables 1 and 2, B-Series). The optimization cost function was based on fitting measures of network frequency, mean phase coherence, and information theoretic measures of integration and complexity, and the parameter sets were validated using measures of synaptic connection probability and strength, as well as network functional connectivity (see Methods section and S3 Fig). Optimizations were conducted separately for each anesthetic level, i.e., parameter values A1/B1 were optimized to data recorded for 0% desflurane concentration, A2/B2 for 2% desflurane, A3/B3 for 4% desflurane and A4/B4 for 6% desflurane.

**Table 1:** Parameter optimization for simulated anesthetic concentrations when performed on 10 different network realizations. A/B-Series describe optimal values determined by the differential evolution algorithm fitting network connectivity parameters obtained when repeating the optimization for 10 total networks. Optimization includes A-Series, when ACh effects are assumed constant and B-Series, when ACh effects are allowed to change with anesthetic concentration. The scaling factors P_x_ scale the effects of synaptic conductances mediated by the x receptor (x = NMDA, GABA and AMPA). A1-A4/B1-B4 denote optimal parameter sets fit to experimental recordings at varying anesthetic concentrations (0%, 2%, 4%, 6% desflurane, respectively). P_AMPA_ is only fit for the 0% anesthetic case A1/B1. Error displayed is SEM.

	P_NMDA_	P_GABA_	P_AMPA_	g_Ks_		$P_{NMDA}$	$P_{GABA}$	$P_{AMPA}$	$g_{Ks}$
**A-Series** (±SEM)					B‐Series(±SEM)				
A1	**1.69**±0.05	**3.92**±0.51	**1.27**±0.07	**0.98**±0.08	B1	1.69±.0.05	3.92±.0.51	1.27±.0.07	0.98±.0.08
A2	**1.41**±0.03	**4.32**±0.44	**1.27 -**	**0.98 -**	B2	1.63±.0.07	5.51±.0.33	1.27‐	1.07±0.03
A3	**1.22**±0.12	**8.13**±1.21	**1.27 -**	**0.98 -**	B3	1.47±.0.06	6.35±.0.94	1.27‐	1.23±0.02
A4	**1.09**±0.09	**10.61**±1.91	**1.27 -**	**0.98 -**	B4	1.36±.0.09	8.17±.1.41	1.27‐	1.17±0.06

**Table 2:** Parameter values for best fit of simulated anesthesia and cholinergic reversal. Parameters from lowest cost fit (cost averaged across anesthetic levels) used to simulate anesthetic effects and cholinergic reversal. A/B-Series describe optimal values of best fit determined by the differential evolution algorithm for network connectivity parameters obtained when ACh effects are assumed constant (i.e., g_Ks_ is constant; A-Series) and when ACh effects are allowed to change with anesthetic concentration (B-Series). P_x_ denotes scaled changes in synaptic conductance’s mediated by the x receptor (x = NMDA, GABA and AMPA) as described in Table 1. AR/BR-Series represent simulated anesthetic reversal, obtained by increasing ACh effects (decreasing g_Ks_ from A4/B4 levels) while keeping all other parameters constant.

	P_NMDA_	P_GABA_	P_AMPA_	g_Ks_		$P_{NMDA}$	$P_{GABA}$	$P_{AMPA}$	$g_{Ks}$
**A-Series**					B‐Series				
A1	**1.64**	**3.98**	**1.22**	**0.97**	B1	1.64	3.98	1.22	0.97
A2	**1.43**	**4.61**	**1.22**	**0.97**	B2	1.57	5.12	1.22	1.10
A3	**1.12**	**8.51**	**1.22**	**0.97**	B3	1.42	6.02	1.22	1.24
A4	**1.07**	**9.41**	**1.22**	**0.97**	B4	1.27	8.13	1.22	1.18
**A-Series Reversal**					B‐SeriesReversal				
AR1	**1.07**	**9.41**	**1.22**	**0.81**	BR1	1.27	8.13	1.22	0.98
AR2	**1.07**	**9.41**	**1.22**	**0.67**	BR2	1.27	8.13	1.22	0.79
AR3	**1.07**	**9.41**	**1.22**	**0.53**	BR3	1.27	8.13	1.22	0.60
AR4	**1.07**	**9.41**	**1.22**	**0.40**	BR4	1.27	8.13	1.22	0.40

In each optimization run we kept the network structure fixed. Particularly, when optimizing across the A-series/B-series we maintained a single network to guarantee that the cost or loss function monotonically decreased across generations. To check for robustness, we optimized parameters for 10 independent network realizations. For each network optimization, the initial pool of parameters seeding the search was kept the same. Table 1 reports mean and standard error of obtained parameter values of the 10 optimization runs. Table 2, on the other hand, represents the best fit optimized parameter set that was subsequently used to identify anesthetic effects on the dynamics of the network.

With synaptic connectivity parameters fixed at their levels corresponding to 6% desflurane concentration, we then simulated the reversal of the anesthetic effects by increasing AÇh effects as mediated by the muscarinic receptor dependent M-type K+ current (specifically, decreasing its conductance g_Ks_; Table 2, AR/BR-Series).

The synaptic connectivity parameter values determined by the evolutionary algorithm mirrored experimentally identified trends of desflurane effects on excitatory and inhibitory synaptic currents [14,29,30,34] (See Methods section).

Specifically, in the A-series parameters, there was a decrease in the effects of NMDA receptor-mediated current while there was an increase in the effect of GABA-mediated current in response to increases in anesthesia (Tables 1 and 2). A similar trend was obtained in the B-series with the added result that decreasing effects of acetylcholine (increasing g_Ks_) correlated to the effects of increased anesthesia except for the change from B3 to B4. Interestingly, the optimization predicted that, in the B-series, to offset the decrease in neuronal excitability due to decreasing ACh level (i.e., increased g_Ks_) with anesthetic concentration, the increase in GABA_A_ synaptic efficacy was smaller than that obtained in the A-series, and similarly, the NMDA synaptic efficacy was systematically higher as compared to the A-series.

**Fig 2:**
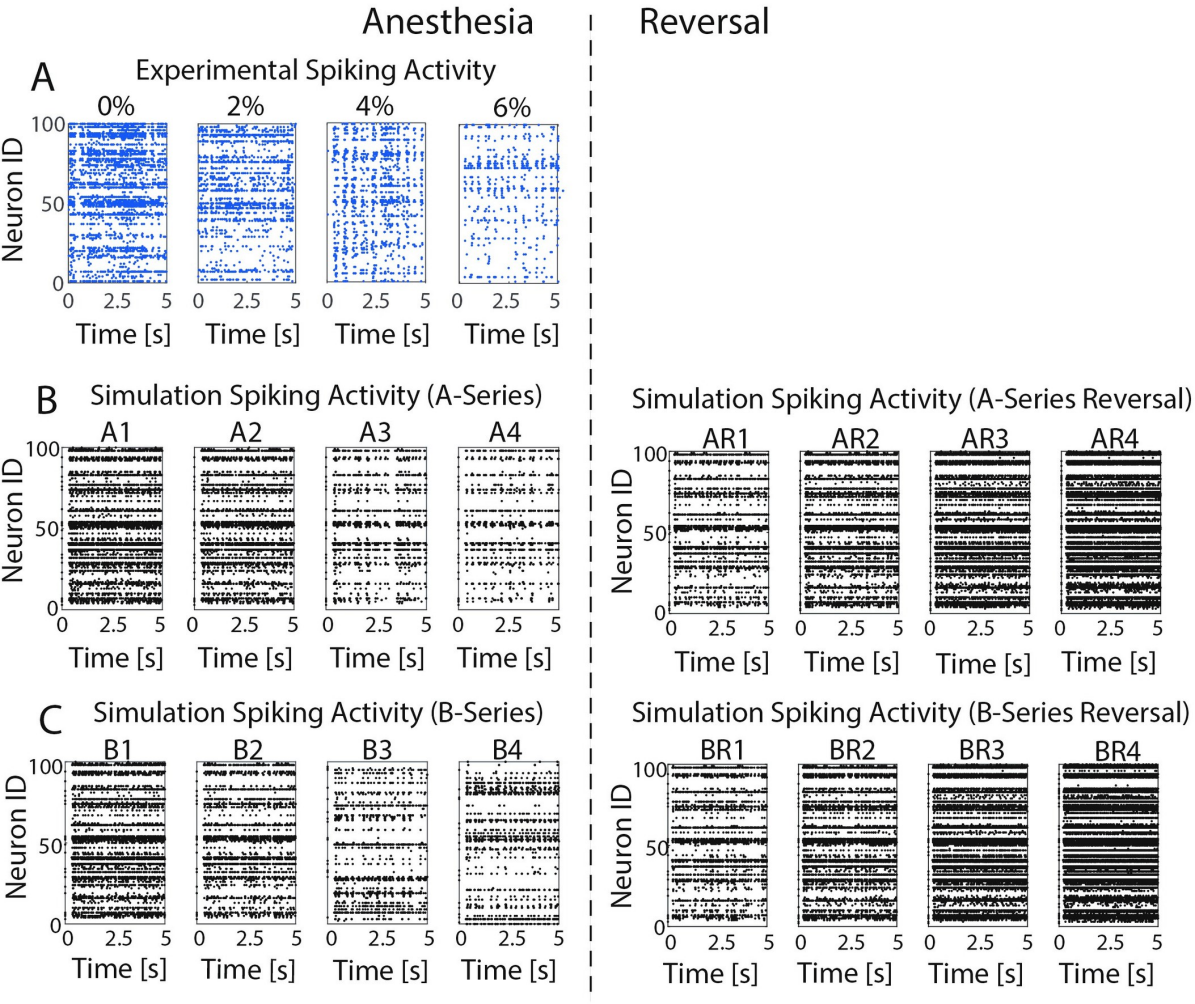
Changes in anesthesia level lead to transitions from high frequency asynchronous to low frequency synchronous spiking patterns. **A).** Raster plots of experimentally recorded neuronal activity in response to changes in desflurane levels. For higher concentrations of desflurane (**6%**), oscillatory synchronous network activity can be seen in spiking dynamics. For lower levels of anesthetic, oscillations are not apparent and asynchronous activity dominates. **B)** Raster plots for simulated anesthetic effects in optimized model networks for constant g_Ks_ (A-Series) and the simulated ACh-induced reversal of anesthetic effects (A-Series reversal). **C)** Raster plots for simulated anesthetic effects in optimized networks with changing g_Ks_ (B-Series) and its reversal (B Series reversal). In both B) and C), simulated anesthetic reversal shows reinstatement of asynchronous from synchronous spiking patterns. Simulation results based on best fit parameters (lowest cost optimization when averaged across anesthesia levels).

Fig 2 shows example raster plots comparing experimental spike timing data collected under the varying desflurane concentrations with model results for the optimized A- and B-series parameter sets, as well as the simulated ACh-induced reversal of anesthetic effects. The model raster plots show similar qualitative trends for increasing simulated anesthetic concentration as the experimental data, specifically spiking patterns change from asynchronous with higher spiking frequencies at simulated 0% desflurane concentration (A1/B1) to a lower frequency, more synchronized firing pattern for simulated 6% desflurane concentration (A4/B4). Furthermore, the simulated reversal via ACh mediated decreases in the M-current (AR1/BR1 –AR4/BR4) reverses those trends.

In the following sections, we analyze how specific characteristics and measures of network dynamics, including frequency distributions and profiles, mean phase coherence, information theoretic measures, and excitatory/inhibitory connectivity probability, computed for the experimental data for progressively increased desflurane levels are reproduced in the optimized model networks. These measures were then computed for simulated increasing levels of cholinergic modulation to analyze the recovery of network dynamics during ACh-induced reversal of anesthetic effects.

### Anesthetic effects on network dynamics and their predicted ACh-mediated reversal

#### Spike frequency decreases in response to anesthesia and recovers in response to decreased M-Current

**Fig 3:**
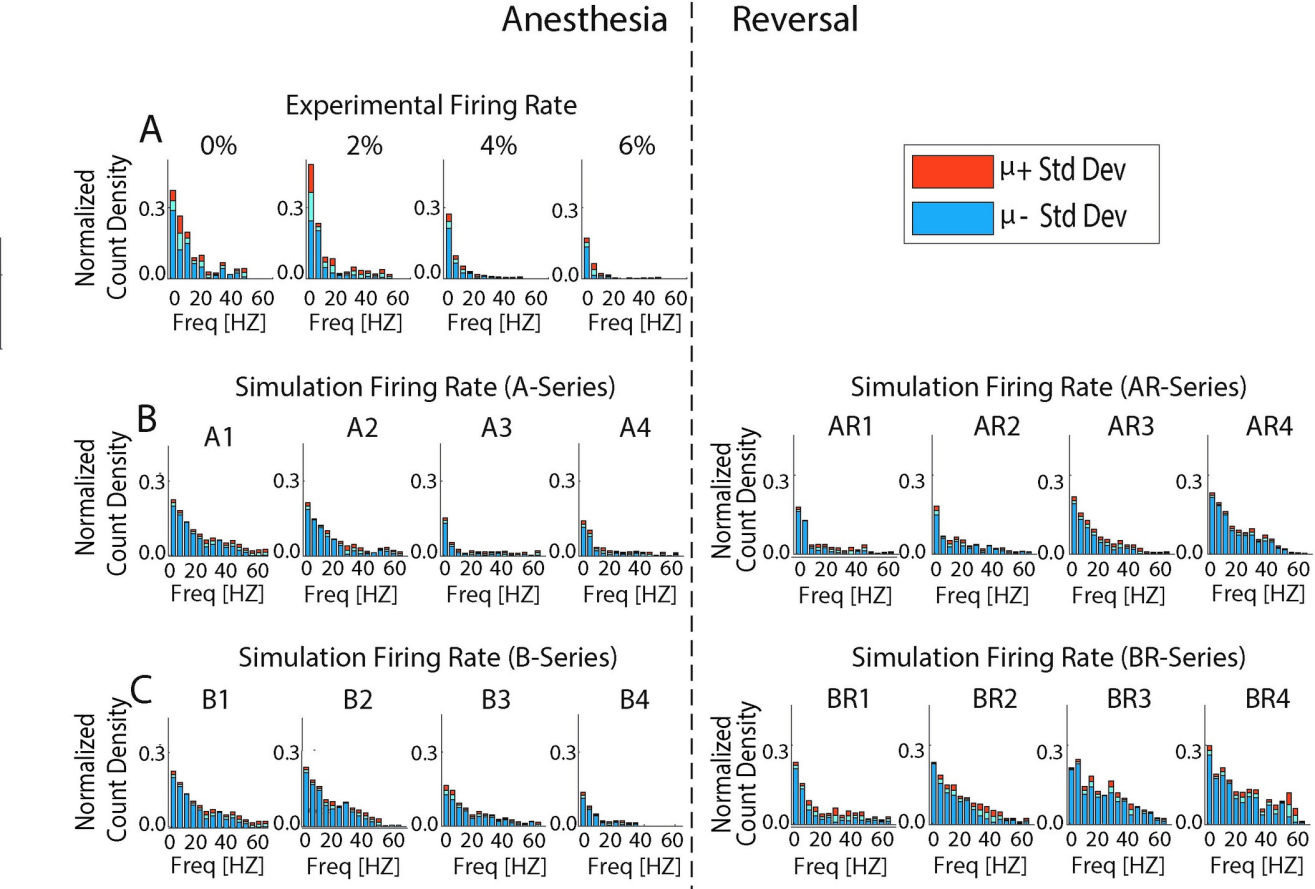
Firing rate distributions for different levels of anesthetic concentration. **A)** Changes in experimentally recorded firing rate distributions under increasing desflurane concentration (0, 2, 4, and 6%) show increased right skewness for the awake state in comparison to anesthetic states. The bins were normalized by the total number of spikes relative to the awake case (0%). **B) and C)** Firing rate distributions in optimized networks for A- (B) and B- (C) series parameter sets. Simulated networks show similar trends in frequency distributions when compared to experiment. The predicted ACh-induced reversal shows reinstatement of the right skew. The bins were normalized by the total number of spikes relative to the awake case A1/B1. Upper/Lower bound show histogram standard error. Results based on lowest cost fit parameters.

**Fig 4:**
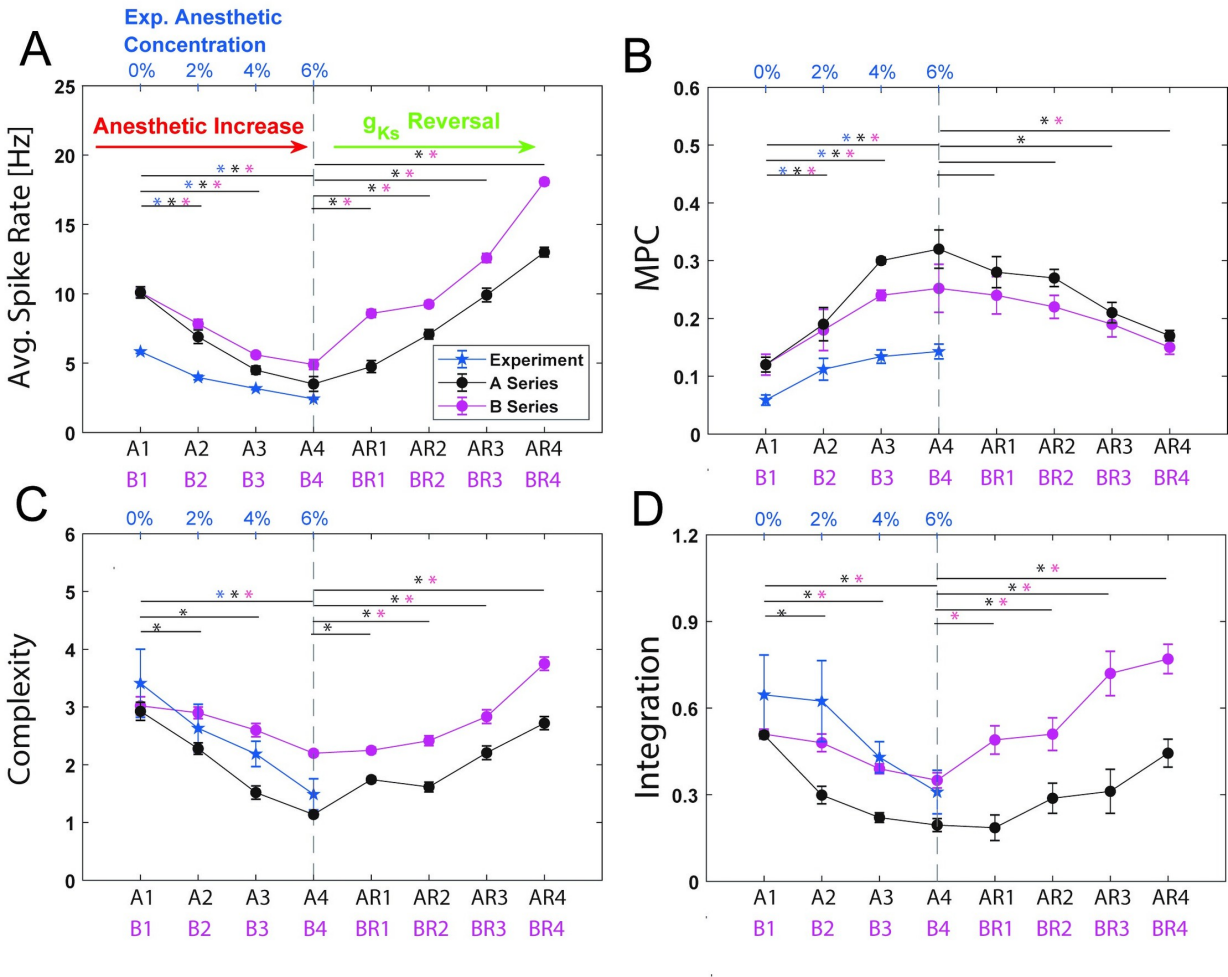
Characterization of anesthetic effects on network dynamics and their simulated ACh reversal for best fit. Measures of network dynamics computed from experimental data and optimized model networks as a function of anesthetic concentration and simulated reversal level: **A)** Average spike rate **B)**. Mean Phase coherence **C)** Complexity C(X) **D)** Integration I(X). A1-AR4/B1-BR4 (x-axis) denote simulated anesthetic concentration levels and reversal states obtained in optimized networks with corresponding parameters listed in **Table 2**. Black line denotes simulations with A-series parameter sets (g_Ks_ constant) and pink line denotes simulations with B-series parameter sets (changing g_Ks_). Blue line (with corresponding axis labels on the top) denotes measures computed from experimental spiking data at different desflurane concentrations. Stars denote significance between initial anesthetic/reversal and subsequent simulations. All calculations were made for 6s intervals and then averaged over 5 intervals. Error bars are +/-SEM based on 10 network realizations. Results from lowest cost fit parameters.

We first characterized the changes in the mean neuronal spike frequency as well as the shape of the neuronal spike frequency distributions as a function of anesthetic level in the optimized model networks (Figs 3 and 4A). We observed that the neurons generally fired less, in both the experimental data and the simulations, as a function of anesthetic concentration. Also, the spread of neuronal firing frequencies decreased significantly with increased anesthetic level, with the loss of the right skew observed in the wake cases (0%, A1 and B1). Spike frequency decreased as a function of desflurane concentration for both parameter series, (A and B series, without and with g_Ks_ changes, respectively), with a similar frequency drop, irrespective of the implemented g_Ks_ changes that affect neuronal excitability in the B-series. In the predicted ACh-induced reversal simulation, the rightward skew in frequency distributions was recovered, and the B-series showed stronger recovery in mean spike frequency as compared to the A-series. This is because, as mentioned above, accounting for cholinergic changes on neuronal excitability under desflurane anesthesia predicts that synaptic changes are less severe. Namely, in the B-series, GABA_A_ synaptic strength was not as high, and NMDA synaptic was not as low compared to the A-series. In the experimental data, anesthesia reduced excitatory firing frequency in a dose dependent manner (p<0.05, correlation test). This was likewise observed in both A- and B-series simulations (r_E_ = -0.97, r_A_ = 0.-95, r_B_ = -0.99, P_E_ <0.0001, P_A_ <0.0001, P_B_<0.0001). Moreover, firing frequency in the awake state showed a significant difference between all subsequent anesthesia states (p<0.0167, Bonferroni) for the experiment as well as for the A- and B-series. Likewise, when comparing firing rates in all of the subsequent reversal states to the highest anesthesia state (e.g. A4 to AR1-AR4) a significant difference was found (p<0.0125, Bonferroni). As anticipated, the reversal simulation had positive firing rate correlations for both A- and B- series (r_AR_ = 0.89, r_BR_ = 0.93, P_AR_ <0.0001, P_BR_<0.0001).

#### Neurons phase lock for increasing anesthesia and decohere with decreased M-Current

In both experimental and simulated results, the common feature was an increase in network synchronization as a function of increased desflurane levels. Mean phase coherence (MPC) measures the consistency of the relative phase that neurons fire with respect to each other thus taking into account non-zero time lag synchrony. Anesthesia increased MPC (Fig 4B) in the data and both simulation series (r_E_ = 0.64, r_A_ = 0.81, r_B_ = 0.71, P_E_ = 0.0052_,_ P_A_ <0.0001, P_B_ <0.0001). Moreover, MPC in the awake state showed a significant difference between all subsequent anesthesia states (p<0.0167, Bonferroni) for the data as well as for the A- and B-series (Fig 4B).

For the anesthetic reversal, increased levels of ACh (i.e. decreased g_Ks_) led to decreases in MPC. The reversal data for experiment and simulation showed an overall positive trend (r_AR_ = -0.50, r_BR_ = -0.52, P_AR_ = 0.0003, P_BR_ = 0.0001); however, a significant difference between the means of individual levels was only present between the highest reversal state and the deepest level of anesthesia for the B-series (B4 and BR4, P<0.0125, Bonferroni) and between the two highest states and the deepest anesthesia for the A-series (A4 and AR4/AR3, P <0.0125, Bonferroni).

#### Information theoretic metrics decrease in response to anesthesia and increase with smaller M-Current

We computed the information theoretic measures network integration (I(X)) and complexity (C(X)) for both experimental data and simulated network activity. Integration is a generalization of mutual information that measures the amount of total entropy of a system that is accounted for by the interactions among its elements. Integration is zero when system elements are statistically independent [35]. Complexity, on the other hand, measures the total entropy loss due to interaction of system elements, or, equivalently, the difference between the sum of the entropies of the individual elements and the entropy of the entire system. Complexity is low for systems with independent elements or with highly synchronous elements.

Integration and complexity displayed similar changes in both the experiment and simulation with increasing anesthetic concentration (Fig 4C and 4D). Both displayed negative trends for simulated anesthesia and positive trends for reversal. For the experiment a statistical difference was only found between 0% and 6% for the complexity (P<0.0167, Bonferroni) while no significant difference was found for experimental integration. For the simulation, the B-series complexity showed significant changes between B1 and B4, the A-series additionally had differences between A1 and all levels of anesthesia complexity (P<0.0167, Bonferroni). Complexity showed significant negative correlation with level of anesthesia for both A and B series as well as the experiment (re = -0.73, r_A_ = -0.96, r_B_ = -0.75, P_E_ <0.0002, P_A_<0.0001, P_B_<0.0001). The reversal simulation complexity displayed a positive linear trend for both AR and BR-series (r_AR_ = 0.76, r_BR_ = 0.62, P_AR_ <0.0001, P_BR_ <0.0001) with significant differences between A4 and all reversal states for the A-series and between B4 and all reversal states with the exception of BR1 for the B-series (P<0.0125, Bonferroni). Consistent correlations were seen for integration (r_E_ = -0.55, r_A_ = -0.82, r_B_ = -0.76, P_E_ = 0.0165, P_A_ <0.0001, P_B_ <0.0001) and its reversal (r_AR_ = 0.47, r_BR_ = 0.76, P_AR_ = 0.0006, P_BR_ <0.0001). For the integration simulation, a significant difference was seen between the awake case and all anesthetic states in the A-series and between the wake and highest anesthetic state for the B-series (P<0.0167, Bonferroni). For the reversal, significant differences were seen between B4 and all reversal states for the B-series and between A4 and all reversal states with the exception of AR1 for the A-series (P<0.0125, Bonferroni).

A difference in trends between the A and B series simulations is indicated by the significantly more precipitous drop in the measures for the A-series with increasing anesthetic level. This could be due to the differences in network connectivity parameters (i.e., NMDA and GABA_A_ synaptic strengths) obtained for the two series. Specifically, lower NMDA synaptic efficacy and higher GABA synaptic efficacy leads to effective disconnection of the neurons in the A-series networks, resulting in lower integration and complexity measures.

Simulated M-current mediated reversal acted to increase both these measures (Fig 4C and 4D; AR/BR series). In the B-series reversal, both measures recovered to values greater than the simulated waking values A1/B1. This was presumably due to the higher NMDA and lower GABA_A_ synaptic efficacies that lead to significantly stronger excitatory interactions between the neurons in the B-series simulations, increasing integration and complexity.

#### Connectivity strength decreases in response to anesthesia and increases with M-Current mediated reversal

We estimated network excitatory and inhibitory synaptic strengths, as well as network excitatory and inhibitory connection probabilities, in the optimized networks and compared them directly to these same measures computed from the experimental data. These excitatory and inhibitory network connectivity measures were computed using cross correlogram analysis as described in the methods section and based on methods estabilished in previous works [14,36,37].

**Fig 5:**
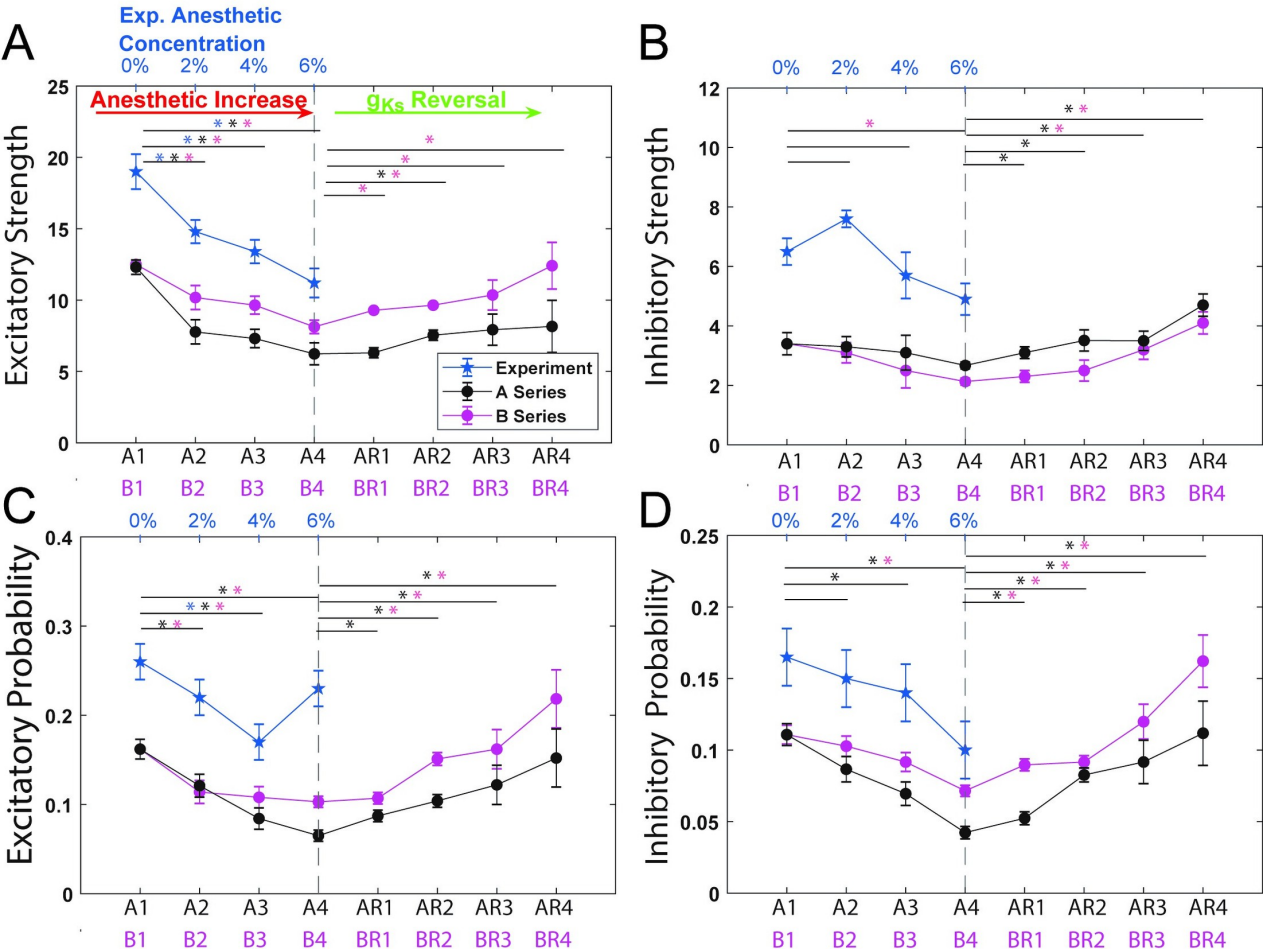
Characterization of anesthetic effects on network connectivity and their simulated ACh reversal. Measures of network connectivity computed from experimental data and optimized model networks as a function of anesthetic concentration and simulated reversal level: **A)** network excitatory connectivity strength, **B)** network inhibitory connectivity strength, **C)** network excitatory connectivity probability, **D)** network inhibitory connectivity probability. A1-AR4/B1-BR4 (x-axis) denote simulated anesthetic concentration levels and reversal states obtained in optimized networks with corresponding parameters listed in **Table 2**. Blue line (with corresponding axis labels on the top) denotes measures computed from experimental data, black (pink) line denotes measures computed from A-series (B-series) network simulations. In these measures, the presence of a significant connection was determined through cross correlogram analysis as described in Methods section. Stars denote significance between initial anesthetic/reversal and subsequent simulations. Error bars of +/- SEM based on 10 network realizations for best fit optimization.

The optimized networks displayed similar decreases in the strength of excitatory network connectivity with increased levels of anesthetic as observed in the experimental data (Fig 5A). When testing for statistical significance via a correlation test we found that anesthesia reduced excitatory strength. The excitatory strength had a significant negative correlation with anesthesia treatment (r_E_ = -0.79, r_A_ = -0.81, r_B_ = -0.77, P_E_ <0.0001, P_A_<0.0001, P_B_ <0.0001). The wake state showed a significant difference between all subsequent anesthesia states (P<0.0167, Bonferroni) for the experiment as well as for the A- and B-series. Excitatory strength in the reversal simulation had positive correlations for the A- and B-series (r_AR_ = 0.43, r_BR_ = 0.46, P_AR_ = 0.0018, P_BR_ = 0.0008). For the reversal, the A-series only showed differences between A4 and AR2 whereas the B-series showed differences across all reversal states (P<0.0125, Bonferroni). Both the A-series and B-series results followed similar trajectories, with the A-series results reporting somewhat smaller excitatory connectivity strength values. This can be due to the fact that the evolutionary algorithm returned significantly lower NMDA efficacy for the A-series, compared to the B-series. On the other hand, excitatory network connectivity probability is very similar for both parameter series as the structural connectivitiy density of excitatory synapses is the same in all model networks (see Methods section). We found a significant correlation in excitatory probability in the A-series and B-series but not in the experiment. A significant difference was found between all subsequent anesthesia states and the wake state (A1/B1) for A- and B-series simulations (P<0.0167, Bonferroni). The experiment, A-series and B-series had negative correlations but only the A- and B-series demonstrated a significant correlation (r_A_ = -0.87, r_B_ = -0.55, P_A_ <0.0001, P_B_ = 0.0053). Differences were found between all reversal states and A4/B4 except between B4 and BR1 (P<0.0125, Bonferroni). The reversal simulation had positive correlations for both A- and B-series (r_AR_ = 0.54, r_BR_ = 0.59, P_AR_ <0.0001, P_BR_ <0.0001).

The experimental data, as well as simulation results for both the A and B series networks, showed decreases in inhibitory network connectivity strength and probability as a function of anesthetic concentration. Inhibitory strength showed negative correlations with increasing anesthetic but with a significant correlation only for the B-series (r_B_ = -0.54, P_B_ = 0.0003). There was only a significant difference between the awake state B1 and B4 (P<0.0167, Bonferroni). The reversal simulation had significant correlations for both A- and B-series (r_AR_ = 0.50, r_BR_ = 0.61, P_AR_ = 0.0002, P_BR_ <0.0001). A significant difference was seen between A1 and all reversal states in the A-series and between B1 and B3, B4 for the B-series (P<0.0167, Bonferroni). This seems a counterintuitive result since GABA_A_ synaptic efficacies increase with desflurane level, and were explicitly modeled as such in our networks. However, this result may be a consequence of decreases in excitatory network synaptic strength and connectivity probability. Namely, inhibitory cells receive less excitatory drive, subsequently firing fewer spikes and, thus, limiting their effect on postsynaptic targets.

Inhibitory probability had significant negative correlations for the simulation A-series and B-series as well as the experiment (r_E_ = -0.49, r_A_ = -0.82, r_B_ = -0.71, P_E_ = 0.021, P_B_ <0.0001, P_B_ <0.0001). Only the simulation showed significant differences between the mean of the wake state and the subsequent anesthetic states. The A-series showed a significant difference between A1 and A3 and A4 while the B-series only showed a difference between B1 and B4 (P<0.0167, Bonferonni). The reversal simulation had positive correlations for both A- and B-series (r_AR_ = 0.67, r_BR_ = 0.57, P_AR_ <0.0001, P_BR_ <0.0001). The reversal simulations showed significant differences between the deepest state of anesthesia and all of the reversal states. This means that there was a significant difference between A1 and AR2-AR4 for the AR-series and B1 and BR2-BR4 for the BR-series (P<0.0125, Bonferonni). Additionally, we observed that the strength of network inhibitory connectivity in the A-series networks was generally stronger than in the B-series networks. This observation agrees with the fact that the GABA_A_ conductance is higher in the A-series parameters than in the B-series. Counterintuitively, network inhibitory connectivity probability was lower and more variable in the A-series networks compared to the B-series networks.

#### Effects of ACh-induced anesthetic reversal on network functional connectivity

The results discussed above report trends observed for measures of average network activity, such as frequency, mean phase coherence, integration and complexity, as well as network connectivity strength and probability. And while M-current mediated reversal reinstated these network-level measures, the measures do not account for recovery of functional connectivity in the network which would contribute to information processing. In this section, we investigate how M-current mediated reversal affects the relative frequency profile of individual neurons with respect to other neurons in the network and also look at effects of reversal on the cellular-level functional connectivity. These measures specifically assess whether the internal dynamic structure of network activity is reinstated during the ACh reversal.

**Fig 6:**
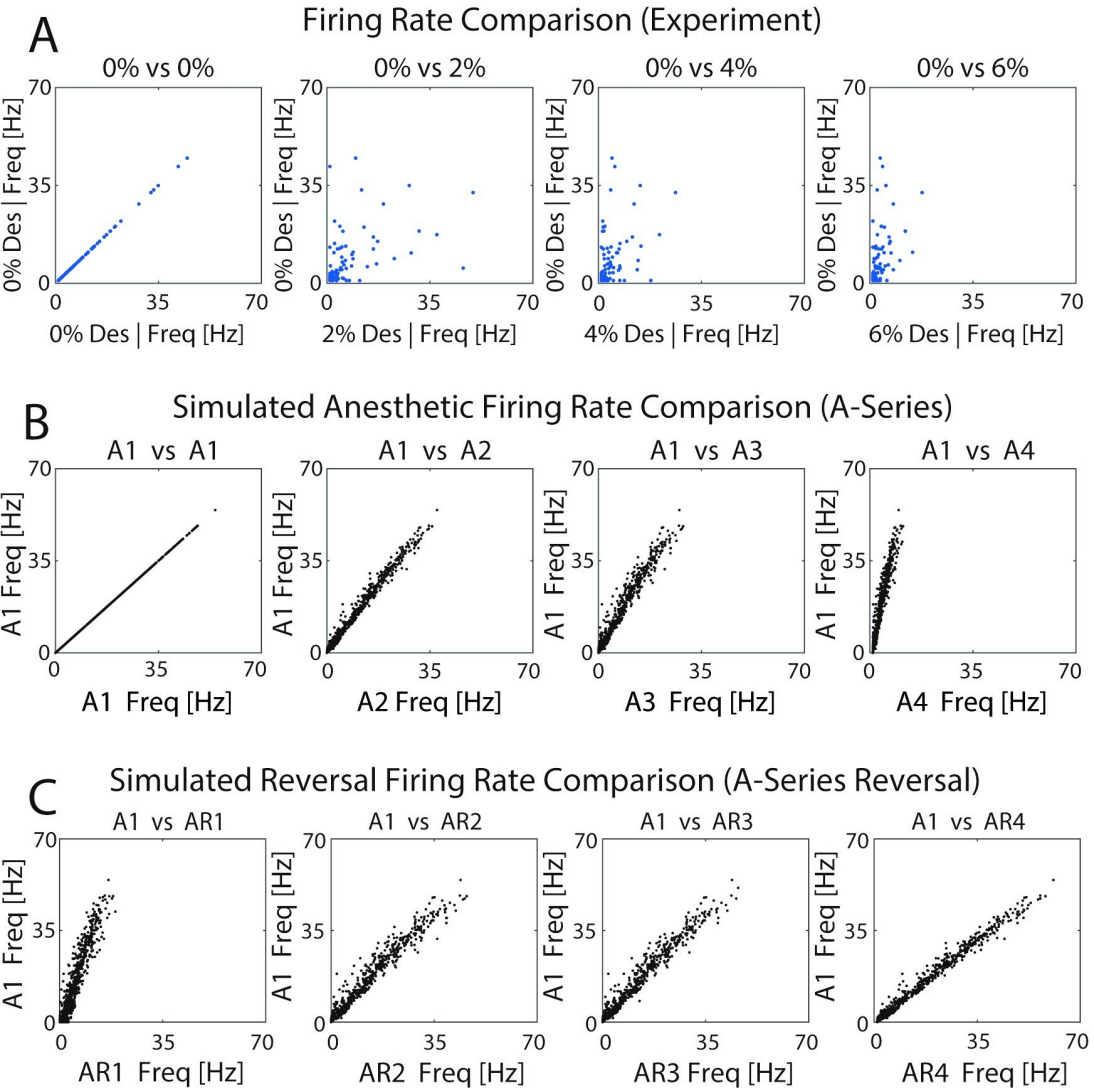
Effects of anesthetic concentration and simulated ACh-induced reversal on relative profiles of neuronal firing frequency. Each panel depicts the firing frequency of each neuron in a given anesthetic/reversal state (x-axis) compared to its firing frequency in the non-anesthetic condition (0% desflurane or A1) (y-axis) **A)** Units recorded in the experimental data; **B,C)** Neurons in A-series optimized networks and reversal.

To accomplish this, we first compared the firing rate of each neuron (or unit) in the experimental data and in the optimized networks at each level of anesthetic concentration to its firing rate in the waking state (Figs 6 and S1). In the figure panels, the x-axis represents firing frequency of individual cells for different anesthetic levels and the y-axis represents the firing frequency for the same cells in the non-anesthetic (0% or A1/B1) conditions. In this figure each plot shows neurons from 10 different optimizations on a single network structure (neurons from 10 different parameters all on same network). Each dot shows the firing rate of a neuron in the 0% on the y axis and the comparison anesthetic level on the x axis. For the experimental data, mutliple units can be potentially detected on a single electrode. This led to potential ambiguity in neurons assigned across different anesthetic levels. To address this issue, neuron identity was based on firing rate in the 0% case. Namely, for units recorded on each electrode, the fastest firing units for 0% anesthesia were given the same ID as the fastest firing units in the 6% case. The results showing an overall linear relationship (Figs 6 and S1) indicates preservation of relative frequency ordering between the neurons. Deflection of the slope of the linear relationship towards vertical indicates the decrease in absolute firing frequency observed for different anesthetic levels.

We observed that, generally, in both experiments and simulation results the relative frequency of the neurons was preserved, i.e. neurons that fired at higher frequencies as compared to other cells in non-anesthetic conditions retained higher firing frequencies at the different anesthetic levels, albeit absolute frequencies decreased. Conversely, neurons that maintained lower firing frequencies (relative to other cells) in the non-anesthetic state continued firing at lower relative frequencies in the anesthetic conditions. Qualitatively similar results were observed for A-series networks (Fig 6) and B-series networks (S1 Fig).

Importantly, during the simulated ACh-induced reversal (AR-series in Fig 6C; BR-series in S1 Fig), the relative relationship between firing frequencies of neurons remained the same, with individual cell frequencies increasing back towards their non-anesthetic values as evidenced by the slope of the linear relationship for higher reversal states tending towards one. This result suggests that individual cells return to roughly the same firing rates during M-current mediated reversal as they exhibited in the simulated waking state. The relationship between firing frequencies had a signficant linear relationship in the simulation (Fig 6B and 6C) with all relationships displaying significant positive correlation (P<0.0001). The experimental relationship (Fig 6A) also had a positive significant relationship (R_0,2%_ = 0.51, R_0,4%_ = 0.46, R_0,6%_ = 0.52, P_0,2%_ = 0.0124, P_0,4%_ = 0.0271, P_0,6%_ = 0.0092).

**Fig 7:**
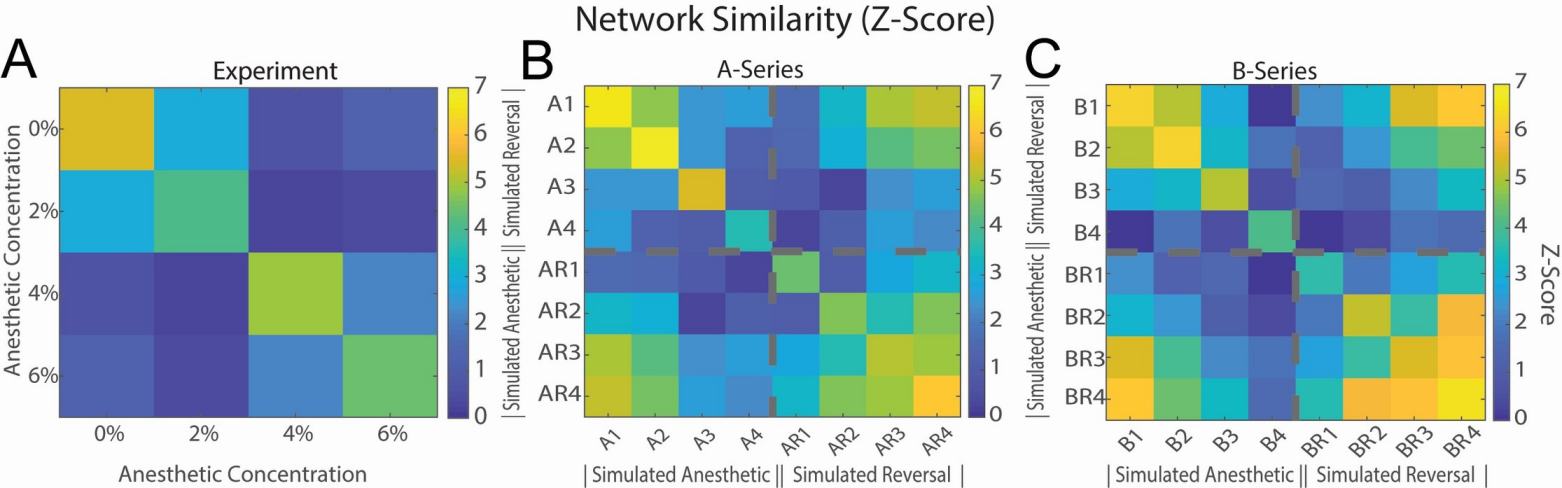
Effects of anesthetic concentration and ACh induced reversal on similarity between cellular functional connectivity. Cosine similarity Z-Score was computed for every pairwise functional connection between neurons. **A)** Experimental functional connectivity was computed between the highest firing neuron for each electrode with similarity computed across different levels of anesthesia. **B,C)** Functional network similarity computed for simulated anesthesia and reversal. Z-Scores were computed comparing the Network similarity to mean and standard deviation of similarities for distributions randomly jittered +/- 5 milliseconds. Each is averaged over ten runs.

Further, to explore detailed changes in cellular-level functional connectivity in the optimized networks, we created functional adjacency matrices from the estimated pairwise excitatory connectivity strengths at all simulated anesthetic and reversal conditions, measured via identification of the peak/trough of the spiking cross correlogram as described in the Methods section. We then calculated the cosine similarities between the created functional adjacency matrices obtained for each anesthetic and reversal level (Fig 7). A cosine similarity of 1 indicates that the functional adjacency matrices are identical, whereas cosine similarity of zero indicates that they are uncorrelated. We then calculated the Z-score of the cosine similarity matrix by comparing the cosine similarity for simulated spike trains with the cosine similarity of connectivity computed from jittered spike trains as described in the Methods. Having a high Z-score indicates how the functional connectivity differs from random. The analysis was performed on experimental data as well as A-series and B-series fits, on all measured excitatory connections. S3 Fig shows example of functional connectivity observed for an individual experiment, simulation and its reversal.

We observed that the functional network similarity matrices, for experimental data as well as model results, became less correlated with each other with increasing anesthetic levels (Fig 7). At the same time, for the model data, M-current mediated reversal resulted in a significant increase in the correlation between the baseline non-anesthetic adjacency matrix (A1 or B1) and the fully reversed functional adjacency matrix (AR4 and BR4). The experimental data had similar behavior with increased similarity between the 0% and 2% anesthesia states when compared to the similarity between 0% and 4%, 6%. The interesting feature of the experimental data is that 6% and 4% had increased similarity when compared to each other than with other anesthetic states (Fig 7). This was not seen in the simulation.

In summary, our model results showed that multiple measures of network connectivity (Figs 5 and 7) increased with ACh-induced simulated reversal suggesting that increases in cellular excitability, mediated by muscarinic effects on the M-current, can reinstate network dynamics dictated by synaptic connectivity.

## Discussion

The goal of this investigation was to simulate the multisynaptic effects of an anesthetic and the modulating effect of muscarinic ACh receptor activation in a neuronal network model. To do this, we applied a computational model of a network of excitatory and inhibitory neurons and used a differential evolution algorithm to fit model parameters to match measures of spiking activity, neuronal connectivity, and network dynamics recorded in the visual cortex of rodents during anesthesia with desflurane *in vivo*.

We first examined if excitatory and inhibitory synaptic changes typically produced by the inhalational anesthetic desflurane led to neural network behavior similar to experimentally observed neuron activity as characterized by various measures including population firing rate, phase coherence, monosynaptic spike transmission, and the information theoretic measures integration and complexity. Second, we investigated if an exogenously induced increase in the level of ACh acting on muscarinic receptors and the M-current could reverse the effect of the anesthetic as suggested by prior behavioral experiments.

### Simulation of the anesthetic effect

We simulated the effect of anesthetic desflurane on the neuronal network by reducing the response of excitatory synapses and facilitating that of inhibitory synapses. General anesthetics commonly potentiate GABAergic synaptic receptor transmission through modification of inhibitory post synaptic potential (IPSP) amplitude and duration, as well as through inhibition of glutamatergic receptor excitatory post synaptic potential (EPSP) amplitude and duration. The relative strength of these effects depends on the class of anesthetic [4,38]. Desflurane inhibits binding at NMDA receptors while potentiating postsynaptic inhibition at GABA_A_ receptors. Some anesthetics, but not desflurane, also suppress AMPA receptors. The effect of anesthetics on nicotinic and muscarinic receptors is more diverse. Some anesthetics also modify the activity of cholinergic neurons projecting to the cortex [23]. Regarding its electrophysiological effects, desflurane has been shown to decrease average spike rate, excitatory and inhibitory monosynaptic transmission, and population measures of neuronal interactions in the cortex [14,39]^.^ These changes in neuronal activity observed *in vivo* have not been directly linked to the corresponding synaptic effects observed *in vitro*.

In our study we found that potentiation of inhibitory GABAergic and inhibition of excitatory glutamatergic NMDA synaptic receptors do indeed lead to graded decreases in population activity and increases in synchronization, as quantified by firing rate and mean phase coherence, as well as measured decreases in integration and complexity. Additionally, we were able to recover changes in functional network connectivity which matched changes seen in literature [25,26]. The simulation results were robust; although only a few of the measures (frequency, MPC, I(X) and C(X)) were used for optimization of model parameters via the differential evolution algorithm, the results held for a wide range of non-fitted measures within limits that produced physiologically reasonable spiking behavior. The correlation test, when giving significance for the experiment and the simulation, gave the same sign of correlation for each metric. Specifically, this validates that our model correctly accounts for the same trends observed in the experiment in response to anesthesia. Moreover, the parameter fits obtained for increasing levels of anesthetic matched in their relative magnitudes to the reported anesthetic induced changes in synaptic efficacy.

### Understanding the mechanism of anesthesia through computational modeling

The cellular mechanism of anesthetic action with respect to loss of awareness has been a subject of intense investigation. Computational models are actively used to make progress in this area of research. Because differing classes of anesthetics elicit different effects on synaptic receptor subtypes, many modeling approaches aim to determine how nuanced changes in receptor binding and synaptic activity lead to changes in neural or electroencephalographic activity. For example, in mean field models, GABAergic and glutamatergic synaptic changes are attributed to a single parameter that maps to different concentrations of general anesthesia [40]. Other modelling approaches seek to understand the mechanism of specific anesthetic agents; for example, the effects of propofol have been studied through the modeling of both GABA_A_ and GABA_B_ amplitude/duration and the effects on cortical synchrony and EEG rhythms [24,41].Enflurane and isoflurane are other commonly modeled anesthetics where the roles of both glutamatergic receptor binding and GABAergic effects are taken into consideration [41–43]. Anesthetic action effected through post synaptic potential (PSP) changes, from a modelling perspective, is a relativity robust explanation supported by its effectiveness across modelling paradigms. These include “mean field” models as well as networks of “integrate and fire”, “Izhikevich “and “HH” neurons, which all show reduced activity and changes to population synchrony when modeling anesthetic effects on synaptic receptors [43–45].

Our study is distinguished from former computational models of anesthetic effects by the independent consideration of the effects on NMDA_R_ and GABA_R_ through PSP changes, as well as of cholinergic influence through changes in the muscarinic M-current. We also used a more biologically realistic log-normal distribution for synaptic weights [46]. Because we had access to experimental spike data, we were able to directly fit our model to empirical data at graded levels of anesthesia and then test our hypothesis regarding cholinergic anesthesia reversal.

### Anesthetic effects on spike synchrony

A common brain signature of general anesthesia is the loss of global functional connectivity between specialized regions of the cortex while local populations show increases in neural synchrony [25,47,48]. Cellular and network mechanisms leading to neural synchrony have been studied extensively in the field of computational neuroscience [49–51]. A set of possible network wide mechanisms are the PING (pyramidal interneuron network gamma) class of mechanisms, where stable, synchronous activity patterns emerge when inhibition periodically shuts down excitation in the network [52–56]. The propensity of neural network synchrony can also depend on intrinsic cellular excitability properties, an example being changes from Type 1 to Type 2 membrane excitability. Type 1 and Type 2 neural excitability describe the well-characterized differences in spike generation dynamics that can generally occur between different types of neurons, and can occur in the same neuron under different pharmacological conditions, such as changing ACh levels. Type 2 dynamics originate from increased competition between depolarizing and hyperpolarizing currents as compared to Type 1 [57]. These differences exemplify themselves in the onset and steepness of firing frequency-input (i-f) curves and the shape of phase response curves (PRCs) which in turn determine synchronization of the networks. Neurons exhibiting Type 1 excitability respond more rapidly with higher firing frequency changes to changing stimulus magnitude as compared to Type 2 cells, and also decreased propensity to synchronize stemming from the shape of their PRC curves [58–60].

Thus, as also discussed below, ACh can play a double edged role in affecting network synchrony via the muscarinic receptor system. On one hand, decreasing levels of ACh during increased anesthesia levels can promote synchrony, as it has been shown that activation of the K+ M-current mediates the transition from Type 1 to Type 2 membrane excitability [56], while on the other hand, the increase of ACh-mediated effects during reversal can offset the decreasing synaptic efficacies with higher cellular responses (increasing steepness of i-f curve). In our modelling results simulated anesthetic effects and M-current mediated reversal, we show that we can evoke transitions between high frequency asynchronous population behavior and low frequency synchronous activity via both mechanisms: by potentiation of IPSPs and inhibition of EPSPs, and ACh-mediated modulation of cell excitability. For higher levels of anesthesia, while both the simulations and the experimental data showed increasing MPC, simulated networks exhibited a larger increase. This could be due to the fact that our model only represents local network interactions, without incorporating the existence of external inputs that could additionally desynchronize network activity leading to decreased MPC. For example, in the visual cortex, there are non-local network inputs possibly preventing a high level of synchronization in the locally recorded network activity, and reflected in lower MPC values in the experimental data. However, overall, our model results demonstrate that it is possible for the population synchronization observed in response to anesthesia to develop in response to changes in PSP alone or to concurrently active cellular mechanisms.

### Predicting anesthesia reversal by ACh

Prior experimental studies demonstrated that the behavioral expression of the anesthetic state can be reversed by stimulating the cholinergic system of the brain by various means *in vivo* and *in vitro* in both humans and animals [21,23,24,61]. To date, no modelling study has attempted to simulate the reversal of neuronal effects of anesthesia by modulating the interaction between cholinergic and other synaptic effects. In this work we demonstrated that ACh limited to act only via blocking the muscarinic slow potassium M-current can reverse the general anesthetic effect on spiking dynamics and population activity, via mechanisms described above. Specifically, we showed that decreasing the influence of the M-current under simulated anesthesia leads to an increase in firing rate and neural interaction measures, showing a population wide reversal of anesthesia-induced synaptic changes. This finding suggests a possible cellular mechanism for the induced reversal of anesthesia effects on PSPs consistent with experimental studies [17,22].

The role of muscarinic ACh receptors in affecting the state of the animal depends largely on the type of general anesthetic used. Desflurane exerts a nonlinear effect on muscarinic ACh receptor activation in a concentration-dependent manner [13]. We also showed that the addition of decreasing acetylcholine influence via the muscarinic pathway during anesthesia (B series) leads to similar reversal endpoints to those with altering NMDA and GABA synaptic changes alone (A series). The choice to model changes in anesthetic ACh influence (B series) in addition to synaptic changes alone (A series) was made to generalize the effects of common inhalational anesthestics which can affect both the cholinergic as well as the glutamatergic and GABAergic pathways (Fig 1). By considering solely the effect of changes on IPSPs via GABA_R_ and EPSPs through NMDA_R_ we show that not only can changes in population activity (firing rate, synchronization and entropy), be accomplished without changes in cholinergic influence but that increasing cholinergic influence alone can reverse these effects. This demonstrates that cortical cholinergic presence has the potential to mitigate the general effects of inhalational anesthesia. In many cases, however, such as for the effects of desflurane, inhalational anesthesia can affect muscarinic and nicotinic ACh receptor binding and for this reason we decided to model the cooperative effects from changes in synaptic EPSP/IPSP and cellular excitability changes via the M-current. In the case of cholinergic reversal, however, this confounded the role of ACh, as the changes in ACh due to anesthesia could be argued to be trivially reversed in the reversal states.

In this study, we used measures of synaptic functional connectivity, computed from average pairwise correlations of neuron spiking, to quantify changes in overall network behavior in both anesthesia and reversal conditions. We showed that the cosine similarity in the functional connectivity matrix increased for the full reversal state when compared to the high anesthetic state. This means that specific neuron to neuron functional connectivity was highly correlated between the awake and reversal states but not the anesthesia states. This suggests that the functional topology of a network can be reversed through a different receptor pathway than is used to achieve the state of anesthesia. Likewise, the population measures of integration and complexity were increased by the cholinergic decrease in M-current. In fact, prior experimental studies showed that muscarinic receptor activation could reverse isoflurane-induced changes in electroencephalogram cross entropy a quantity related to brain functional complexity presumed to be associated with the conscious state [22,62]^.^

In the past, anesthesia reversal has been achieved by a variety of drugs and methods of administration in experimental studies. For example, microinjection of nicotine into the thalamus led to the recovery of the righting reflex in rodents anesthetized by sevoflurane [21], and a similar reversal from isoflurane was observed in response to microinjection of histamine into the basal forebrain [63]. Unlike general anesthesia, however, the mechanisms for induced reversal may be specific to the type of anesthetic agent used. An example of this can be seen when comparing the effects of the GABA_A_ antagonist, gabazine, on the effects of propofol as well as ketamine [64]. The application of gabazine led to wake-like responses when rats were sedated with propofol, which acts through potentiation of GABA_A_ receptors, but gabazine was ineffective when used during administration of ketamine, which has been known to act through modulation of NMDA receptors. These previous studies suggest that the phenomena of induced reversal can be demonstrated in controlled rodent studies, but a similar effect has been suggested in human studies [65]. Another example is the clinical case where a patient’s use of Ritalin, a central nervous system stimulant, required an increase of general anesthetic dose for sedation [66]. In rodents, Ritalin was found to cause emergence from sedation induced by isoflurane [67].

Our results predicting cholinergic recovery of neuronal population dynamics, inter-neuronal functional connectivity and complexity lends support to the evidence that the brain state altered by anesthesia is at least partially reversible. In clinical use, the effects of anesthesia can linger after the drug is no longer administered [68]. For this reason, there are both translational and phenomenological motivations to investigate induced recovery from anesthesia. Our study gives insight into the synaptic and network mechanisms by which central nervous system changes caused by anesthesia can be mitigated by the administration of a functional agonist.

### Limitations and directions for future work

We recognize a few limitations of this study. First, our model was based on random connectivity between E and I cells, instead of on a detailed representation of a specific neural circuit. Other modeling studies included thalamocortical interactions [69,70] or a cortical macro structure aimed at understanding how a multilayer architecture can influence the effects of anesthesia-induced changes in synaptic strength [69,71–73]. We argue that, for a first approximation, a generic random model is sufficient because little is known about the identity of brain regions that are responsible for mediating the anesthetic action, particularly with respect to the suppression of consciousness. We compared our model predictions to data obtained from visual cortex, which may not be the primary site of anesthetic action to suppress consciousness. It can be argued, however, that the experimentally observed changes in neural firing rates, coherence, connectivity, complexity, and other related measures are probably sufficiently general to describe the mechanism of synaptically induced network effects we intend to understand. We corroborated our simulation of anesthetic effects with corresponding data obtained from experiments investigating the effects of desflurane on cortical neuron firing *in vivo*. Regarding the anesthesia reversal, additional experiments will need to be performed to determine the cholinergic effects on spiking behavior under anesthesia. This could serve as a possible avenue for future work to validate our current predictions and to provide further insight into the cellular and network-wide mechanisms of induced anesthetic reversal. Former studies to-date are limited to the demonstration of cholinergic stimulation-induced behavioral reversal [23] and reinstatement of awake-like LFP oscillations [74].

Of course, to understand the full effect of anesthesia on the observed behavior of a live animal may require the modeling of additional effects in multiple brain regions including widespread cortical areas, thalamus, subcortical and brainstem arousal centers, to mention a few. Although other models of anesthetic mechanism have incorporated thalamocortical interactions [69,72], none have simulated anesthetic reversal and still fall short of modeling corresponding behavioral effects. Additionally, general anesthetics have secondary effects on voltage-gated and ligand-gated channels, two-pore potassium channels, and other targets that were not represented in our model [4,13,38]. Inclusion of these additional effects could provide a more nuanced simulation with potentially closer fit to experimental data. Moreover, our model predicts the reversal of cortical dynamics solely from the modulation of the M-current, which only accounts for some changes induced by acetylcholine. We recognize that there are many cholinergic affects not accounted for in our model, however despite the missing details, the success of our simulations suggests that our model likely captured essential mechanistic elements of anesthetic action and its cholinergic reversal.

### Conclusion

In summary, we demonstrated that experimentally observed changes in neural activity and functional connectivity caused by desflurane could be computationally reproduced by modulating synaptic efficacy according to the known synaptic effects of the anesthetic. Additionally, we showed that by modulating the M-current alone, the effect of anesthesia on neural activity and functional connectivity in the network could be at least partially reversed. In the future, more comprehensive models that take into account cortical architecture, thalamocortical interactions and a broader array of cellular mechanisms will help to fully understand the complex roles of synaptic modulation in producing the observed neuronal network and behavioral effects of anesthesia.

## Methods

### Experimental data

Experimental results were based on the analysis of data collected in previous studies; for an in depth description refer to the original study [75] Briefly, rats were surgically implanted with a multishank, 64 contact microelectrode array in the visual cortex (V1). After a post-surgery recovery period, they were placed in a cylindrical anesthesia chamber for administration of inhalation anesthetic. Desflurane was applied in the sequence of 8, 6, 4, 2, and 0% inhaled concentrations for 45 to 50 min at each level. Neural activity was recorded during the duration of the experiment and subsequently processed to extract multiunit spiking information. For this study, we analyzed unit spiking activity collected during the 0, 2, 4 and 6% desflurane exposure sessions and chose not to model 8% due to the confounding mechanism leading to the burst suppression phenomena normally present at this level of anesthesia [76,77].

### Neuron modeling

**Fig 8:**
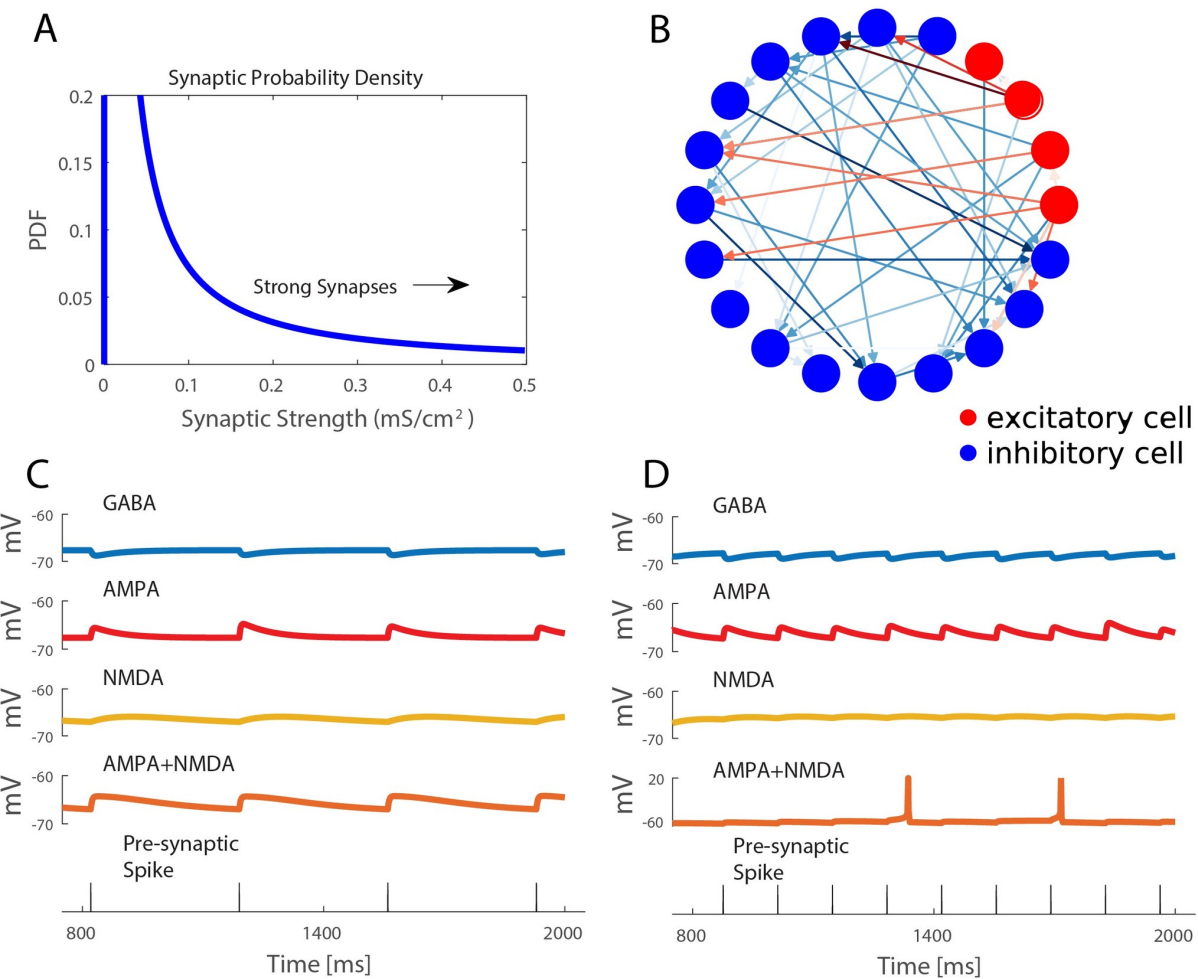
Network structure is populated by lognormal distributed random connection strengths. **A).** Synaptic strengths in model networks varied according to a lognormal distribution with a minority of connections being mediated by strong synaptic strengths, while weak synaptic strengths constitute majority of connections **B)** Simulated network consists of 200 inhibitory and 800 excitatory cells connected randomly with 10% probability. Connection color reflects the log of synaptic strength. **C, D)** Postsynaptic potential time courses in response to synaptic currents mediated by different receptors. Excitatory currents are modeled with both AMPA and NMDA mediated currents. Bottom panel shows timing of presynaptic spikes, for simplicity both inhibitory and excitatory presynaptic neurons are shown with the same spike times.

The computational neuron model was based on the standard Hodgkin–Huxley modeling paradigm with parameters chosen to better account for the firing characteristics of cortical pyramidal neurons as established in previous studies [55,59,78]. In summary the neurons were modeled with coupled differential equations to account for nonlinear changes in voltage as a response to both tonic input and non-periodic input from connected neurons. A visual reference for the voltage response can be seen in Fig 8. For the remainder of the section a description of the model will be given for completeness including the parameters used in the model and structure of the governing equations.

Excitatory and inhibitory neurons are modeled using the Hodgkin-Huxley formalism with parameters selected based on a model that emulated properties of both cortical pyramidal neurons and inhibitory interneurons [79–81]. The neuron model contained sodium, delayed rectifier potassium, slow M-Type potassium and leak currents as described in the following equations:

$$\frac{dV}{dt}=-g_{Na}m_{\infty }^{3}h(V-E_{Na})-g_{Kd}n^{4}(V-E_{k})$$

$$-g_{Ks}z(V-E_{k})-g_{L}(V-E_{L})+I_{noise}-I_{syn}+I_{DC}$$ (1)

$$\frac{dX}{dt}=\frac{X_{\infty }(V)-X}{\tau_{X}(V)}for\;x=\{h,n,z\}$$ (2)

$$m_{\infty }(V)=\frac{1}{1+e^{\left(\frac{-V-30}{9.5}\right)}}$$ (3)

$$h_{\infty }(V)=\frac{1}{1+e^{\left(\frac{V+53}{7.0}\right)}}$$ (4)

$$n_{\infty }(V)=\frac{1}{1+e^{\left(-\frac{V+30}{10}\right)}}$$ (5)

$$z_{\infty }(V)=\frac{1}{1+e^{\left(-\frac{V+39}{5.0}\right)}}$$ (6)

$$\tau_{h}(V)=0.37+\frac{2.78}{1+e^{\left(\frac{V+40.5}{5.0}\right)}}$$ (7)

$$\tau_{n}(V)=0.37+\frac{1.85}{1+e^{\left(\frac{V+27}{15}\right)}}$$ (8)

$$\tau_{z}(V)=75$$ (9)

In the above, *V* is the membrane voltage while *m*, *n*, *h* and *z* represent the unitless gating variables of the ionic current conductances. *I*_*syn*_ is the synaptic current input to the cell from other neurons in the network and has units of *μA*/*cm*^2^. *I*_*noise*_ is a noise input consisting of randomly occurring brief current pulses with average frequency of 0.1 Hz, a duration of 2 ms and strength of 4 *μA*/*cm*^2^. This noise input was sufficiently strong to generate an action potential in the absence of any other inputs. *I*_*DC*_ is a biasing constant current input of -0.77 *μA*/*cm*^2^. *E*_*Na*_, *E*_*K*_ and *E*_*L*_ are the reversal potentials for sodium, potassium, and leak currents, respectively, set to *E*_*Na*_ = 55 mV, *E*_*K*_ = −90 mV, *E*_*L*_ = −60 mV.

This neuron model, with the slow M-type K+ current, was developed to model the muscarinic-receptor effects of acetylcholine in cortical pyramidal neurons [57]. The properties of this neuron model when *g*_*Ks*_ = 0 mS/cm^2^ describe a neuron under high levels of acetylcholine while *g*_*Ks*_ = 1.5 mS/cm^2^ represents a low acetylcholine state.

### Network design

We constructed E-I networks with 800 excitatory and 200 inhibitory neurons (Fig 8B). The choice of excitatory-inhibitory ratio was based on the physiological ratio of 4 to 1 cells found in cortex [82]. Neurons were connected randomly with 10% probability. Synaptic strengths followed a log normal distribution, as suggested to occur in cortical networks (Fig 8A) [46]. The distribution was defined by parameters *μ* = −20.0, *θ* = 9.4, and characterized by the equation:

$${PDF}_{Log}(X)=\frac{1}{x\theta \sqrt{2\pi }}\left(e^{-\frac{{\left(\ln x-u\right)}^{2}}{{2\theta }^{2}}}\right)$$ (10)

*μ* and *θ* are defined such that they are the mean and standard deviation of the logarithm of x if the logarithm of x was normally distributed. This connectivity distribution was chosen such that ~0.2% of excitatory connections would elicit an action potential in a post-synaptic cell in the absence of other inputs for our parameter values representing the wake state. The value of 0.2% was determined by experimental data in which cross correlogram analysis showed a 0.2% “strong” connection probability among a local population of neurons [14].

Synaptic currents mediated by AMPA, NMDA and GABA_A_ receptors were included in the network such that excitatory synaptic currents were given by *I*_*exc*_ = *I*_*AMPA*_+*I*_*NMDA*_ and inhibitory synaptic currents by *I*_*inh*_ = *I*_*GABA*_. All synaptic currents were modeled with a double exponential function of the form

$$I_{X}=P_{x}{B_{x}V}_{0.5}g_{log}(e^{-\frac{t-t_{spike}}{\tau_{Xs}}}-e^{-\frac{t-t_{spike}}{\tau_{Xf}}})(V-E_{x})$$ (11)

where X indicates the receptor type (AMPA, NMDA or GABA_A_), t_spike_ is the time of the presynaptic spike and g_log_ is the synaptic conductance drawn from the lognormal distribution.

Reversal potential *E*_*x*_ was set at −75 mV for inhibitory synapses and 0 mV for excitatory synapses [83]. The term g_o_ will be used to refer to *B*_*x*_ *V*_0.5_ *g*_log_.Time constants *τ*_Xs_ and *τ*_Xf_ governed the fast rise and slow decay of the synaptic current and were set as follows:

$$t_{AMPAf}=t_{NMDAf}=t_{GABA_{A}f}=0.2\;ms$$ (12)

$$t_{AMPAs}=3.0\;ms{,\;t}_{NMDAs}=200.0\;ms,\;t_{GABA_{A}s}=5.5\;ms$$ (13)

The NMDA synaptic conductance was additionally gated by the post-synaptic voltage [84,85] described by the additional pre-factor *B*_*x*_:

$$B_{AMPA}=B_{GABA_{A}}=1$$ (14)

$$B_{NMDA}(V)=\frac{1}{1+e^{-\frac{V+10}{3.57}}}$$ (15)

Fig 2C and 2D illustrates time courses of the synaptic currents. Additionally, to account for event-to-event variability, a variability pre-factor *V*_0.5_, randomly chosen uniformly from [0.5, 1], modulated the synaptic current induced by each pre-synaptic spike. Finally, the scaling factors *P*_x_ simulated anesthetic effects on synaptic conductances. Values of *P*_x_ for each receptor type were optimized to fit multiple measures of network dynamics for each level of anesthesia. Values are listed in Tables 1 and 2 that show average parameter values for optimizations performed on ten different network realizations, and the specific parameter values used for the presented analysis of results, respectively.

### Measures and metrics

We use several different measures to quantify the changes between network states and dynamics under different levels of anesthesia observed in the experimental data and simulated in the neural network models.

### Integration and interaction complexity

We computed the information theoretic measures Complexity C(X) and Integration I(X) to quantify changes in the entropy of the network [35]. I(X) is a generalization of mutual information that measures the amount of total entropy of a system that is accounted for by the interactions among its elements. I(X) is zero when system elements are statistically independent [35]. C(X) measures the total entropy loss due to interaction of system elements, or, equivalently, the difference between the sum of the entropies of the individual elements and the entropy of the entire system. C(X) is low for systems with independent elements or with highly synchronous elements.

**Fig 9:**
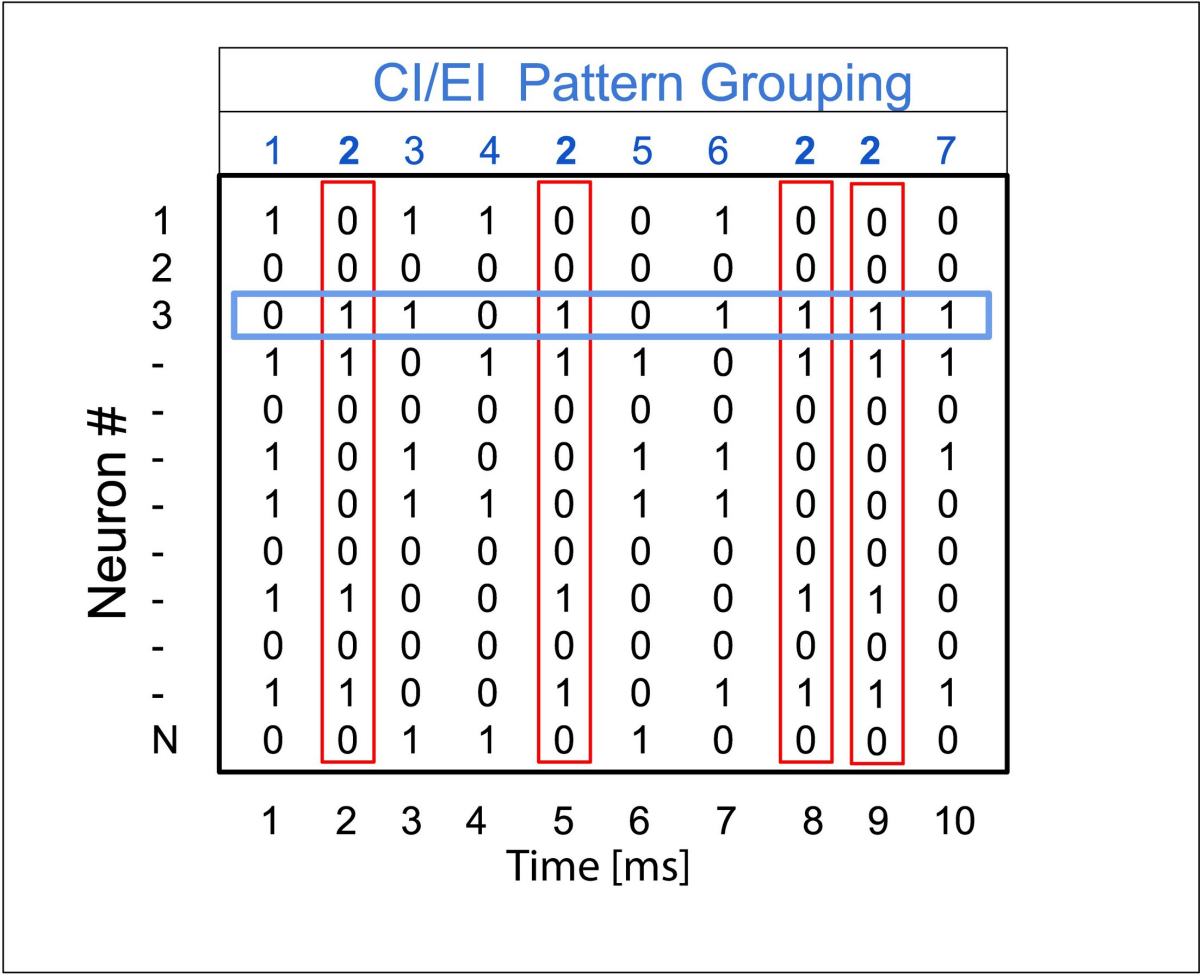
Binned spike patterns for complexity and integration measures. To compute entropy metrics complexity (C(X)) and integration (I(X)), spike trains were binned in 1 ms bins. H(X) in Eqs (16)/(17) is computed according to unique patterns associated with column vectors (red vectors) while H(X_i_) is the entropy associated with a single neuron spike train (blue vector).

To compute these measures, the total spiking activity from an experimental recording or a network simulation was partitioned into patterns by binning spike trains into 1 ms time bins and constructing vectors for each time bin containing a 1 at the neuron index if the neuron spiked within that time bin and a 0 if there was no spike (columns in Fig 9). The set X of unique vectors, representing patterns of spiking activity within a bin, that occurred across the data set were identified. Additionally, discretized spike vectors *X*_*i*_, *i* = 1,…,*N*, were constructed for each cell (rows in Fig 9).

To compute integration and complexity only a subset of neurons were considered. 60 neurons were selected at random from both the experimental data and the simulation. I(X) and C(X) were computed by taking 3 random intervals of 6s, computing the measure on each set of intervals, and then averaging the measure outcomes across the three sets.

Integration was computed as

$$I(X)=\sum_{i=1}^{N}H(X_{i})-H(X)$$ (16)

where *H*(*X*_*i*_) = −∑_*k*_*p*_*k*_*logp*_*k*_ is the entropy based on the probability of a spike occurring in the *i*^*th*^ cell, and *H*(*X*) = −∑_*j*_*p*_*j*_*logp*_*j*_ is the entropy based on the probability of occurrence of a spike pattern vector.

Complexity was computed as

$$C(X)=H(X)-\sum_{i=1}^{N}H(X_{i}|X-X_{i})$$ (17)

Here, *H*(*X*_*i*_) is the entropy of the spike train belonging to neuron i while *H*(*X*) is the entropy of the set of spike vector for the entire interval. *H*(*X*_*i*_|*X*−*X*_*i*_) is the conditional entropy where *X*_*i*_ is the new spike vectors neglecting the *i*^*th*^ unit and is conditioned on the spike train of the *i*^*th*^ unit. The metric is discussed greater detail in original study [35].

### Mean phase coherence

We computed mean phase coherence to quantify the average phase relation between spike times of pairs of neurons in experimental recordings and network simulation. The pairwise mean phase coherence is given by

$$\sigma_{i,j}=\left|\frac{1}{N}\sum_{k=1}^{n}\left(\exp\left(i2\pi \frac{t_{j,k}-t_{i,k}}{t_{i,k+1}-t_{i,k}}\right)\right)\right|$$ (18)

where *t*_*j*,*k*_ is the time of the *k*^*th*^ spike of the *j*^*th*^ neuron and *t*_*i*,*k*_, *t*_*i*+1,*k*_ are times of successive spikes of the *i*^*th*^ neuron. Network mean phase coherence is the average of *σ*_*i*,*j*_ over all pairs of neurons [86].

For two neurons *i* and *j*, the mean phase coherence is 1 when the spike times of neuron *j* always occur at the same relative phase in the cycle defined by two subsequent spikes of neuron *i*. Conversely, pairwise mean phase coherence is zero when spikes of neuron *j* occur at random phases of the neuron *i* spike cycle for the entire set of neurons *i* spike times, due to averaging of phases.

### Functional connectivity probability and strength

**Fig 10:**
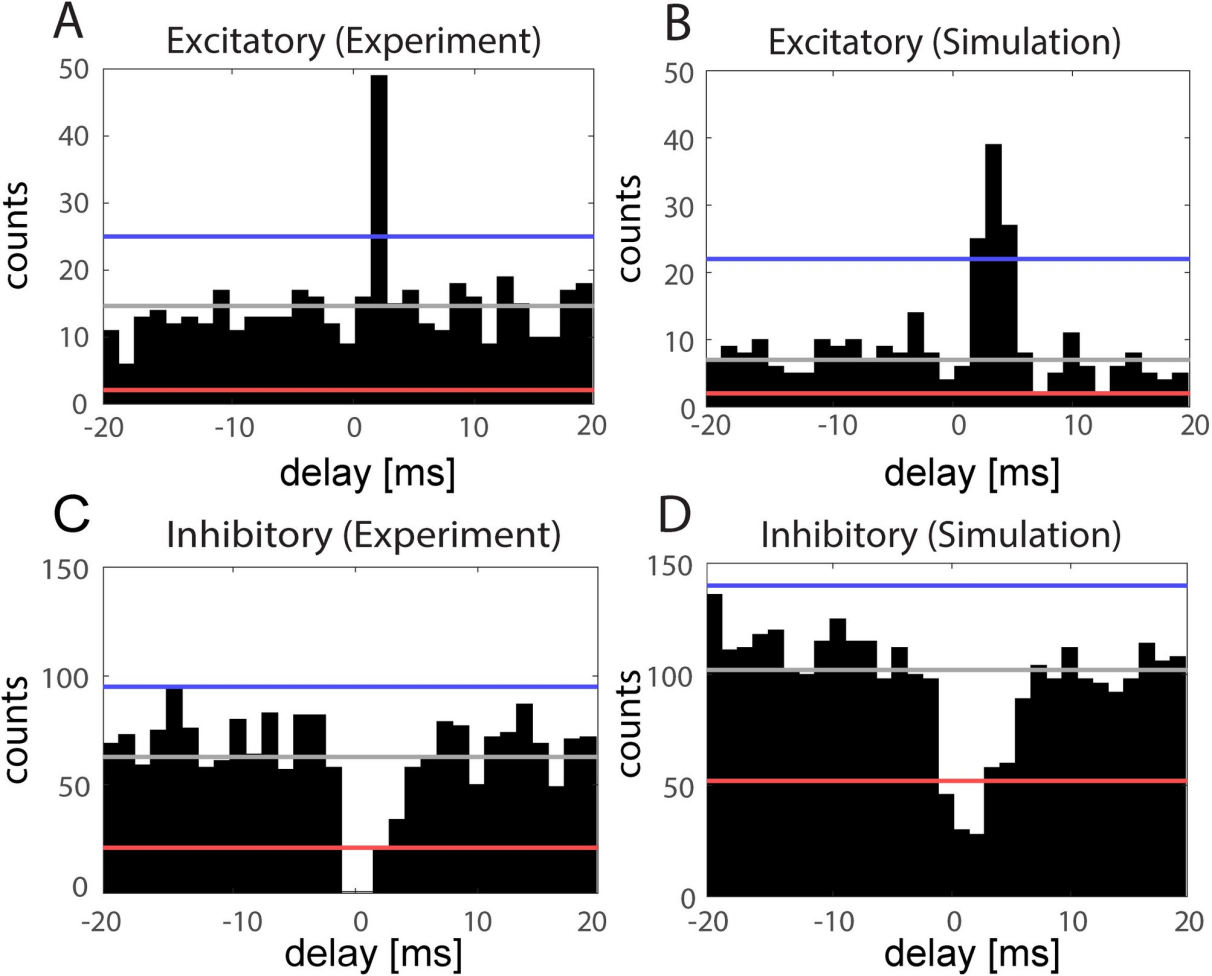
Cross Correlogram computes coincident spike relations by summing relative spike times of reference and comparison neurons. **. A-D)**. Cross correlograms between example pairs of “reference” and “comparison” cells, centered at spike times of the “reference” cell, from the experimental recordings (left column) and simulated networks (right column). Significance bands were computed from a jittered data set of “comparison” cell spike times (gray line = mean of jittered data set, red line = excitatory significance, blue line = inhibitory significance, see text). **A-B)** Example cross correlograms showing significant excitatory connections between cell pairs. **C, D)** Example cross correlograms showing significant inhibitory connections between cell pairs.

Functional connectivity probability and strength were determined through cross correlogram analysis on spike trains [36] between pairs of neurons with minimum average spike rate of 1 Hz. Since experimental recordings contained on average ~60 eligible units, these measures for the simulated networks were computed based on spike trains of 60 eligible neurons. For each pair of cells, spike trains were segmented into 40 ms intervals centered on each spike of the designated “reference” cell of the pair and discretized into 1.3 ms bins. Cross-correlations of discretized segments between the “reference” and “comparison” cell for every “reference” cell spike were summed to form cross correlograms (Fig 10).

Significance of correlations was determined by comparison to a constructed “jittered” dataset. The jittered data set was formed by randomly “jittering” spike times of the “comparison” cell by [–5, 5] ms and then computing the cross correlogram. This was repeated by 100 times to for the jittered data set. The global confidence band for excitatory (inhibitory) connectivity was computed by taking the 97% confidence interval associated with the global peak (trough) of the jittered data set. A significant connection was determined when the standardized peak (trough) of the original cross correlogram was greater (less) than 2 times the 97% confidence interval when measured from the mean (blue/red) [14,36,37]. The standardized peak was formed by dividing the peak amplitude by jittered mean and standard deviations of the cross correlogram.

Excitatory connectivity strength was determined by taking the difference in the peak height within 0 and 5.2 ms (first four bins) and the jittered mean and dividing it by the jittered standard deviation. The inhibitory strength was computed in a similar manner by looking at the trough of the cross correlogram within 0 and 5.2 ms.

### Parameter optimization

**Fig 11:**
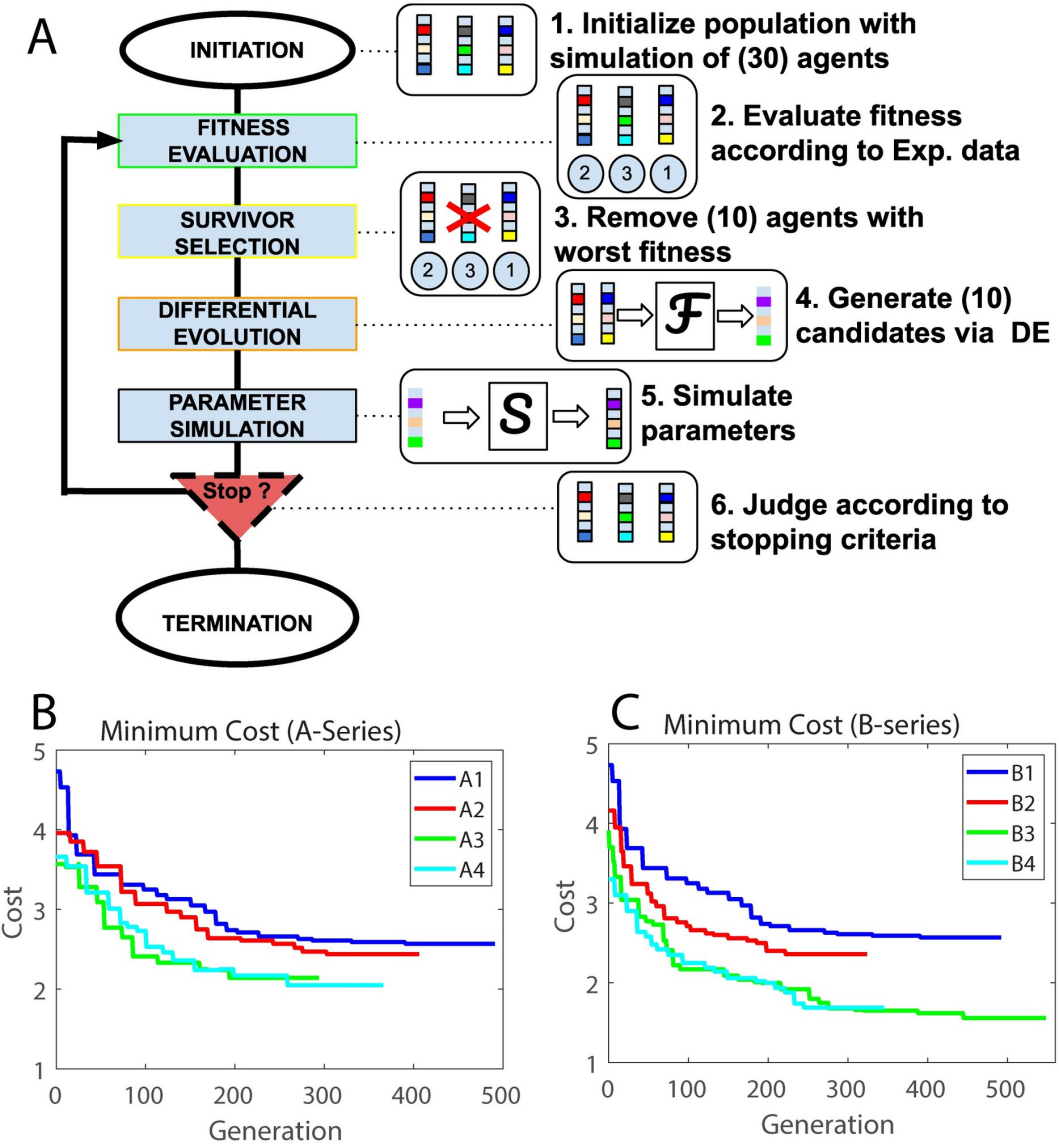
Parameter search fine-tuned through Differential Evolution algorithm. **A).** Evolutionary algorithm procedure, differential evolution, was used to optimize model parameters. For each generation, 10 agents (parameter sets) with the highest cost function from the population of 30, were chosen for replacement. Algorithm was repeated until stopping criteria of 100 generations without change in lowest cost function value across the population was met. **B,C)** Example optimization cost of lowest cost parameters across the population for each generation in the A-series (B) and B-series (C). Population A1/B1, A2/B2, A3/B3 and A4/B4 were optimized to experimental data from the 0%, 2%, 4% and 6% anesthetic cases, respectively. The optimizations for A1 and B1 were identical. In the A-series (A2-A4), *P*_*NDMA*,_ *P*_*GABA*_, were optimized and in the B-Series (B2-B4), *P*_*NDMA*_, *P*_*GABA*,_ *g*_*Ks*_ were varied.

Network model parameters were optimized using an evolutionary algorithm to fit measures of network frequency, mean phase coherence, integration and complexity computed from the experimental unit spiking data collected during the 0%, 2%,4% and 6% desflurane exposure sessions. The optimized parameters were the synaptic conductance scaling parameters *P*_*AMPA*_, *P*_*NMDA*_, *P*_*GABA*_ (A-series) and, additionally to those, the maximal conductance of the M-type K+ current *g*_*Ks*_ (B-series). The algorithm is similar to typical differential evolution procedures [87,88]. Briefly, from a population of 30 agents (parameter sets), at each generation the 10 agents with highest cost function values were replaced with 10 new parameter sets constructed by an evolutionary algorithm described below (Fig 11A). The stopping criteria was 100 generations without change in the lowest cost function (*L(X)*) value across the population of 30 agents. The stopping criteria was chosen as it supports a finite run time in stochastic search and has been used in similar implementations [89,90].

The initial population of 30 parameter sets representing the 0% anesthetic state was chosen from the 256 parameter sets generated by assigning parameter values from the following parameter scan: *P*_*AMPA*_, *P*_*NMDA*_∈{0.5, 1.0, 1.5, 2.0}, *P*_*GABA*_∈{2.5, 5.0, 7.5, 10.5} and *g*_*Ks*_∈{0.3, 0.7, 1.1, 1.5} *mS*/*cm*^2^. We initially searched for boundary parameters that achieved balanced network dynamics and followed this by a the above parameter scan within those bounds. The parameter scan was then followed by our algorithmic fitting procedure. Model networks with fixed connectivity structure and synaptic strength *g*_0_ values were simulated with each parameter set for 20 s and frequency, mean phase coherence, integration, and complexity measures were computed based on spiking activity excluding the initial 1s, to avoid initial transients. The cost or loss function, *L*(*X*), based on these measures, *x* = frequency, MPC, I(X) and C(X), compared values computed from simulations, *x*_*sim*_, and experimental data, *x*_*exp*_, at 0% anesthetic state as follows:

$$L(X)=\sum_{x}m_{x}$$ 19

where

$$m_{x}={\left(\frac{x_{exp}-x_{sim}}{x_{exp}}\right)}^{2}$$ (20)

Here, the 20 lowest cost parameter sets were kept and each parameter value was randomly varied uniformly by 10% of its value to avoid duplicate values. The final 10 parameter sets were then constructed using the differential evolution algorithm.

Similar to typical differential evolution procedures [87,88] we set a cross over probability CR = 0.8 and had a variable differential weight DW that was randomly varied between [0,^2^]. From the subpopulation of 20 parameter sets, 10 randomly chosen sets, *a*^*k*^ (*k* = 1,…,10), formed the basis for 10 newly created sets, *e*^*k*^ (*k* = 1,…,10). For each set *a*^*k*^, 3 different sets *b*^*k*^, *c*^*k*^ and *d*^*k*^ were chosen that were different from *a*^*k*^ and each other. Then, for each element *i* = 1,…,4 in the set, a random number *ρ*_*i*_ from the uniform distribution [0,^1^] was chosen. If *ρ*_*i*_ was less than CR, a new parameter value $e_{i}^{k}$ was generated as $e_{i}^{k}=b_{i}^{k}+DW(c_{i}^{k}-d_{i}^{k})$; otherwise $e_{i}^{k}=a_{i}^{k}$.

This was done for each element in the new agent and was repeated until 10 new agents were created. After this was done the 10 new agents were simulated and then the 30 total parameters were evaluated for their cost. The 10 with the highest cost (worse fit) were then rejected and the process was repeated.

We performed 2 parameter optimizations, A-Series and B-Series, to parse out potentially different effects of anesthetic modulation on synaptic conductances only (A-series) and of combined modulation on synaptic conductances and cholinergic effects (B-series) (Fig 11B and 11C). In both scenarios, populations A1/B1 were the result of optimizing *P*_*AMPA*_, *P*_*NMDA*_, *P*_*GABA*_, *g*_*Ks*_ to the experimental 0% anesthetic case. In the A-series, *P*_*NMDA*_, *P*_*GABA*_, were optimized while in the B-series, *P*_*NMDA*_, *P*_*GABA*_, *g*_*Ks*_ were optimized to the 2%, 4% and 6% anesthetic cases. Optimizations for the 6% anesthetic case, A4/B4, were initiated from parameter values constrained by experimental reports of 20% average decrease in NMDA-mediated synaptic signaling and 40% increase in GABA-ergic synaptic signaling under desflurane [29,91]. These initial values were randomly varied uniformly by +/- 5% to generate variability in the event of parameter convergence. In the optimizations for the 2% and 4% anesthetic cases, A2/B2 and A3/B3, respectively, the initial population for A2/B2 was A1/B1, and the initial population for A3/B3 was A4/B4.

### Simulation of ACh reversal

To validate robustness of the parameter optimization, we ran our optimization for 10 network realizations, keeping the network structure fixed for all anesthetic levels. The average and error (SEM) for the optimized parameters across these 10 networks is shown in Table 1. Table 2 lists the parameter values with the lowest cost function for one of these optimization runs that we used in our model analysis. Simulated cholinergic reversal (AR1-AR4/BR1-BR4) was modeled by decreasing the value of g_Ks_ from the values in A4/B4 to 0.4 mS/cm^2^ such that there were 4 values in the reversal series.

### Statistical analysis

The effects of desflurane and ACh-modulated M-current were tested using RM-ANOVA with the level of intervention as fixed factor on each of the metric for both experimental data and the simulation results. When the effect of the treatment was significant, the individual effects were further examined using individual paired t-tests with Bonferroni correction at *α* = 0.0167 for testing the anesthesia effect (four levels) and *α* = 0.0125 for testing the reversal effect (five levels). To compare trends, additional tests with linear regression were done on the experimental data as well as the A-series/B-series and the cholinergic reversal results, at *α* = 0.05. Statistical analyses were conducted in Excel.

### Functional connectivity analysis

Functional connectivity was determined via cross-correlogram analysis where the connectivity strength (significance) was determined by comparing the peak within 0 to 5 ms lag of the cross-correlogram to the jittered mean and standard deviation of the cross-correlogram. Mean was formed from mean of 100 jittered correlograms and the standard deviation from the jittered means [36,37]. The connectivity strength was recorded for each pair wise connection and then used to determine the cosine similarity between the two simulations by computing the dot product of the pairwise connectivity strengths for different anesthesia levels. This was converted to a Z-Score by comparing average cosine similarity between two anesthesia levels (10 network average) to the cosine similarity between two anesthesia levels created in the same manner from jittered time-series (jittered time lag peak compared to jittered mean and standard deviation).

### Simulations

Custom C++ code was developed for numerical simulations which was run on the Greatlakes High Performance Cluster. For the evolutionary algorithm each model simulation was run for 20s. The stopping criteria was met when the lowest minimum cost remained unchanged for 100 generations (last 100 iterations of cost curve in Fig 11). To check the robustness of the current parameter set, 10 additional generations were run with model simulations of 80s and an increased crossover probability (CR = 0.9). We detected no change in the minimum cost parameter set. For the results shown in Figs 3–5 each simulation was simulated for 150000 ms. The length of this runtime was necessary to result in enough spike times to calculate metrics based on cross correlograms. Results in Figs 4 and 5 are for 10 simulation runs in which network connectivity was randomized across runs but maintained for the different simulated anesthetic levels. In this way, each of the 10 simulation runs corresponds to a unique simulated experiment. On each run the voltage and gating variables were subject to random initial conditions independent of the network seed. On initialization V was uniformly varied between [–72, –32] mV, n between [0.2, 0.6], z between [0.2, 0.3] and h between [0.2, 0.6] while m was initialized at 0 for all runs. The equations were integrated using the 4^th^ order Runge-Kutta method.

## Supporting information

S1 Fig B-Series effects of anesthetic concentration and simulated ACh-induced reversal on relative profiles of neuronal firing frequency. Each panel depicts the firing frequency of each neuron in a given anesthetic/reversal state (x-axis) compared to its firing frequency in the non-anesthetic condition (B1) (y-axis) **A,B)** Neurons in B-series optimized networks and reversal.
(TIF)

S2 Fig Error cost analysis for the lowest cost fit. Error cost breakdown shows subcost for each metric of example fit. For each generation, 10 agents (parameter sets) with the highest cost function from the population of 30, were chosen for replacement. Algorithm was repeated until stopping criteria of 100 generations without change in lowest cost function value across the population was met and performed on single network.
(TIF)

S3 Fig Functional Connectivity for Experiment and Simulated Anesthesia and Reversal. **A).** Example of experimental functional connectivity for 0%-6% anesthesia. Overlap in connectivity can be seen for all concentrations. **B)** Example A-Series functional connectivity. Higher connectivity is seen for A1 and decreases with increasing simulated anesthesia. Common connections between all anesthetic states can be observed. **C)** Example of AR Series functional connectivity. Low connectivity is seen for AR1 and increases with g_Ks_ reversal. Single network/experiment shown in each case.
(TIF)
