# Supplementary figures and images for: Modeling cortical synaptic effects of anesthesia and their cholinergic reversal
Source: PLoS Comput Biol. 2022 Jun 23;18(6):e1009743. doi: 10.1371/journal.pcbi.1009743 (PMC9258872; doi:10.1371/journal.pcbi.1009743)

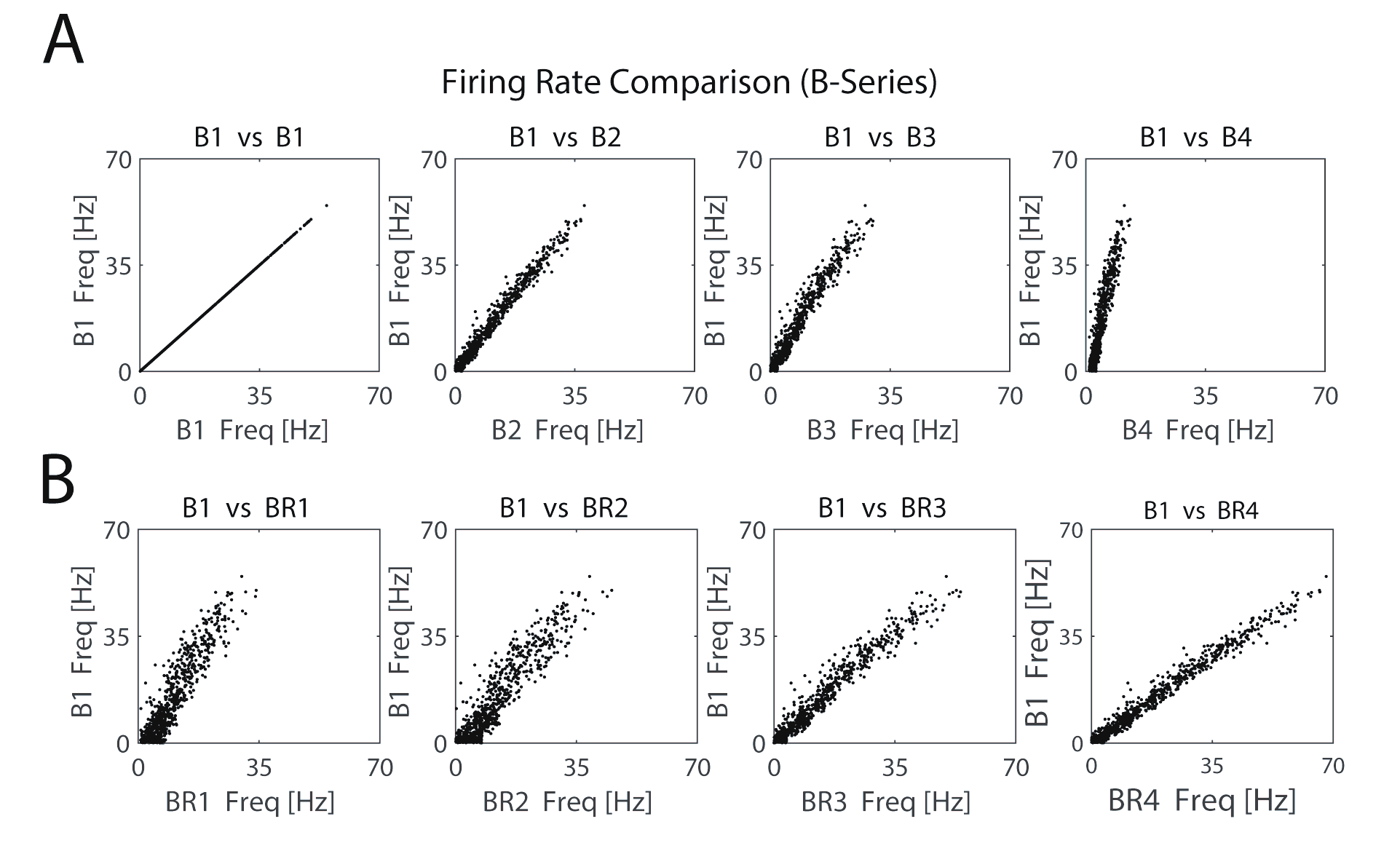

Supplement: S1 Fig — Each panel depicts the firing frequency of each neuron in a given anesthetic/reversal state (x-axis) compared to its firing frequency in the non-anesthetic condition (B1) (y-axis) A,B) Neurons in B-series optimized networks and reversal. (TIF) [file pcbi.1009743.s001.tif]

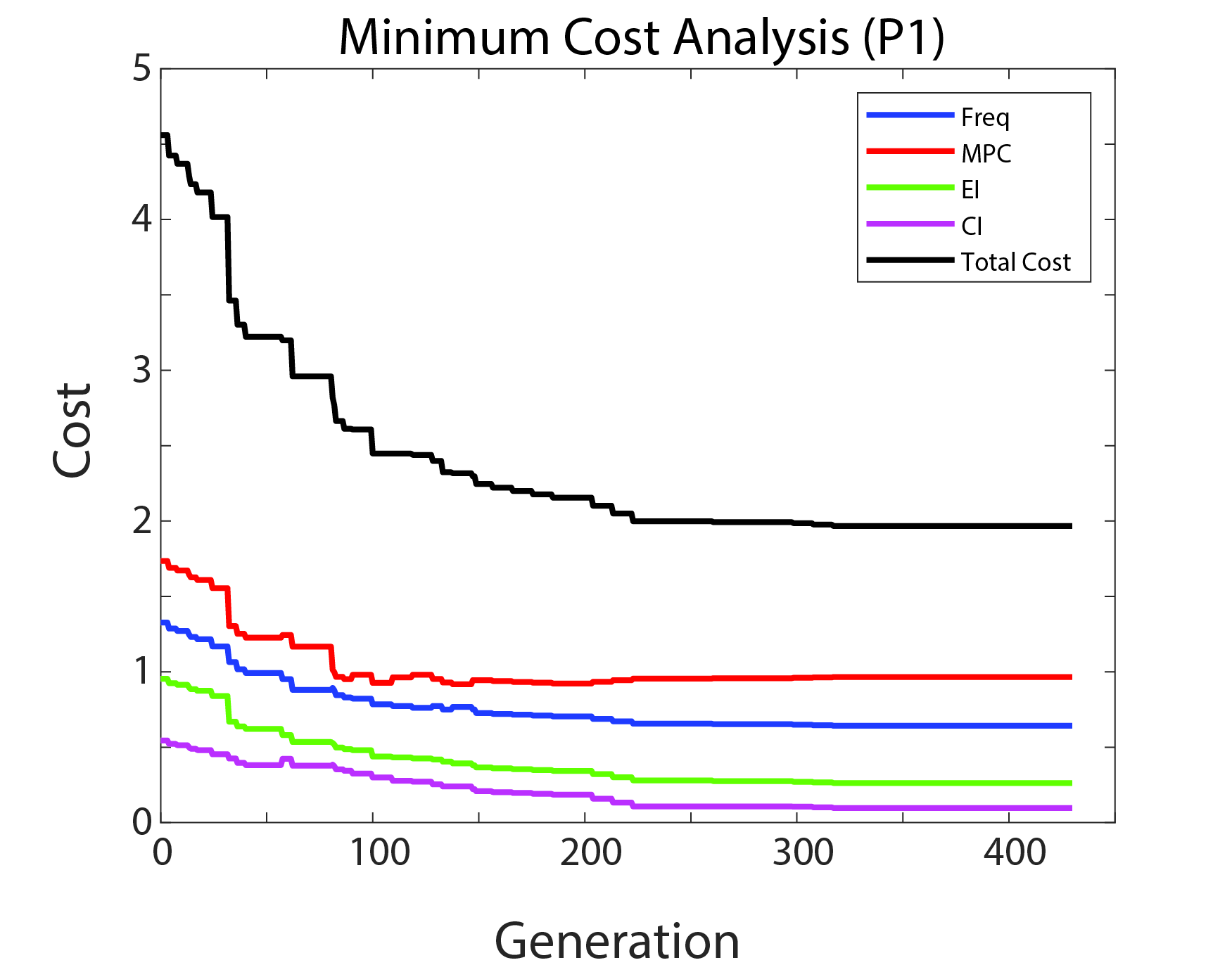

Supplement: S2 Fig — Error cost breakdown shows subcost for each metric of example fit. For each generation, 10 agents (parameter sets) with the highest cost function from the population of 30, were chosen for replacement. Algorithm was repeated until stopping criteria of 100 generations without change in lowest cost function value across the population was met and performed on single network. (TIF) [file pcbi.1009743.s002.tif]

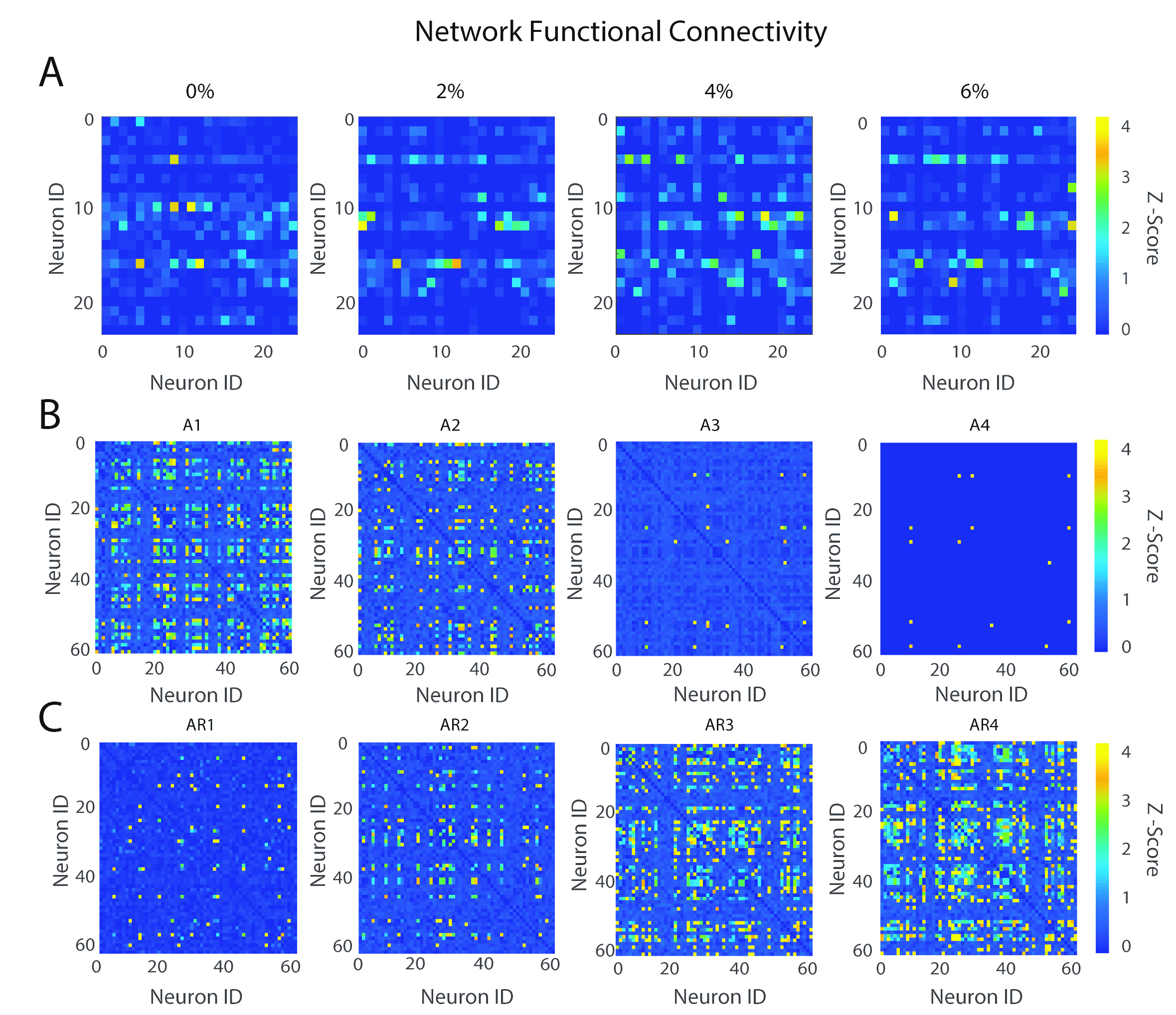

Supplement: S3 Fig — A). Example of experimental functional connectivity for 0%-6% anesthesia. Overlap in connectivity can be seen for all concentrations. B) Example A-Series functional connectivity. Higher connectivity is seen for A1 and decreases with increasing simulated anesthesia. Common connections between all anesthetic states can be observed. C) Example of AR Series functional connectivity. Low connectivity is seen for AR1 and increases with gKs reversal. Single network/experiment shown in each case. (TIF) [file pcbi.1009743.s003.tif]
